# Vascular Morphogenesis in the Context of Inflammation: Self-Organization in a Fibrin-Based 3D Culture System

**DOI:** 10.3389/fphys.2018.00679

**Published:** 2018-06-05

**Authors:** Beate M. Rüger, Tanja Buchacher, Alexander Giurea, Bernd Kubista, Michael B. Fischer, Johannes M. Breuss

**Affiliations:** ^1^Department of Blood Group Serology and Transfusion Medicine, Medical University of Vienna, Vienna, Austria; ^2^Turku Centre for Biotechnology, University of Turku and Åbo Akademi University, Turku, Finland; ^3^Department of Orthopedics, Medical University of Vienna, Vienna, Austria; ^4^Department of Health Sciences and Biomedicine, Danube University Krems, Krems an der Donau, Austria; ^5^Department of Vascular Biology and Thrombosis Research, Center of Physiology and Pharmacology, Medical University of Vienna, Vienna, Austria

**Keywords:** neovascularization, inflammation, 3D fibrin matrix, synovial tissue, mesenchymal stromal cells, self-organization

## Abstract

**Introduction:** New vessel formation requires a continuous and tightly regulated interplay between endothelial cells with cells of the perivascular microenvironment supported by mechanic-physical and chemical cues from the extracellular matrix.

**Aim:** Here we investigated the potential of small fragments of synovial tissue to form *de novo* vascular structures in the context of inflammation within three dimensional (3D) fibrin-based matrices *in vitro*, and assessed the contribution of mesenchymal stromal cell (MSC)-immune cell cross-talk to neovascularization considering paracrine signals in a fibrin-based co-culture model.

**Material and Methods:** Synovial tissue fragments from patients with rheumatoid arthritis (RA) and inflammatory osteoarthritis (OA) were cultivated within 3D fibrin matrices for up to 4 weeks. Cellular and structural re-arrangement of the initially acellular matrix were documented by phase contrast microscopy and characterized by confocal laser-scanning microscopy of topographically intact 3D cultures and by immunohistochemistry. MSC-peripheral blood mononuclear cell (PBMC) co-cultures in the 3D fibrin system specifically addressed the influence of perivascular cell interactions to neo-vessel formation in a pro-inflammatory microenvironment. Cytokine levels in the supernatants of cultured explant tissues and co-cultures were evaluated by the Bio-Plex cytokine assay and ELISA.

**Results:** Vascular outgrowth from the embedded tissue into the fibrin matrix was preceded by leukocyte egress from the tissue fragments. Neo-vessels originating from both the embedded sample and from clusters locally formed by emigrated mononuclear cells were consistently associated with CD45^+^ leukocytes. MSC and PBMC in co-culture formed vasculogenic clusters. Clusters and cells with endothelial phenotype emerging from them, were surrounded by a collagen IV scaffold. No vascular structures were observed in control 3D monocultures of PBMC or MSC. Paracrine signals released by cultured OA tissue fragments corresponded with elevated levels of granulocyte-colony stimulating factor, vascular endothelial growth factor and interleukin-6 secreted by MSC-PBMC co-cultures.

**Conclusion:** Our results show that synovial tissue fragments with immune cell infiltrates have the potential to form new vessels in initially avascular 3D fibrin-based matrices. Cross-talk and cluster formation of MSC with immune cells within the 3D fibrin environment through self-organization and secretion of pro-angiogenic paracrine factors can support neo-vessel growth.

## Introduction

Neovascularization plays an important role throughout postnatal life during regenerative processes after tissue and organ damage by ischemia or injury, in the course of acute or chronic inflammation and during auto-immune diseases. Neo-vessel formation is mainly accomplished by angiogenesis—the expansion of a pre-existing vascular network by endothelial cell sprouting—and is assisted by a process called postnatal vasculogenesis where precursor cells from the bone marrow are mobilized into the circulation, home to sites of active angiogenesis and differentiate into mature endothelial cells (Masuda and Asahara, 2003). Key cellular players of the neovascularization process in an inflammatory setting are immune cells, including neutrophils, macrophages and lymphocytes recruited to perivascular sites (i.e., perivascular niches) together with endothelial progenitor cells (EPC), as well as resident endothelial- and stromal cells. The leakage of fibrinogen into the perivascular space during inflammatory processes creates a microenvironment suitable for these cell types to interact with each other directly or indirectly through the secretion of paracrine factors (Davalos and Akassoglou, 2012). Cytokines act in concert within complex networks, where pro-angiogenic factors amplify the process of inflammation (Aplin et al., 2006) and continuous pro-inflammatory signals promote the process of neovascularization providing evidence for a regulatory intercommunication between inflammation and neovascularization in pathological situations (Costa et al., 2007). Further complexity is added by the pleiotropic effects and redundancy of most cytokines involved in neovascularization and inflammation. Interleukin (IL)-6 has both pro- and anti-inflammatory properties (Scheller et al., 2011) and can indirectly cause an increase in vascular permeability and neovascularization by inducing the expression of vascular endothelial growth factor (VEGF) (Cohen et al., 1996; Tzeng et al., 2013), a cytokine playing a well-known key role in both physiological and pathological angiogenesis (Ferrara et al., 2003). Both VEGF and granulocyte-colony stimulating factor (G-CSF) can contribute to postnatal neovascularization by mobilizing bone marrow-derived EPC (Asahara et al., 1999; Powell et al., 2005), and G-CSF has been shown to modulate the expression of leukocyte adhesion molecules (Sugimori et al., 1999; Suzuki et al., 2002), activate endothelial cells (Bussolino et al., 1989) and enhance angiogenesis (Natori et al., 2002). G-CSF is induced by inflammatory stimuli, and depending on context plays either beneficial or detrimental roles. In mice endogenous G-CSF is a critical mediator of acute and chronic inflammatory arthritis (Lawlor et al., 2004). Beneficial effects of systemic G-CSF administration, however, have been reported in human inflammatory bowels disease associated with changes in memory cell cytokine production (Mannon et al., 2009).

A large range of *in vitro* models of both angiogenesis and vasculogenesis have used fibrin matrices as scaffold to study the mechanisms of endothelial cell assembly into microvascular networks (reviewed in Morin and Tranquillo, 2013). Amongst various endothelial cell (EC) types, human umbilical vein EC (HUVEC) and human dermal microvascular EC have been studied either as monoculture or in co-culture with support cells, such as fibroblasts, smooth muscle cells, mesenchymal stromal cells (MSC), and pericytes (Morin and Tranquillo, 2013). Both stromal cells from the bone marrow and adipose tissue can, when co-cultured *in vitro*, support HUVEC organization into vascular-like structures, which, however, lack a lumen (Verseijden et al., 2010), and adipose-derived stem cells form vascular-like structures when co-cultured with late outgrowth endothelial cells derived from peripheral blood (Holnthoner et al., 2015). These studies provide important information primarily about blood vessel assembly, but they do not take into account the well-recognized connection between neo-vessel formation and inflammation in pathological situations, as *in vivo* blood vessels are surrounded by connective tissue, which contains stromal cells, immune cells and extracellular matrix (ECM)-bound signaling molecules.

Excessive neo-vessel formation is a common feature of many chronic inflammatory disorders including rheumatoid arthritis (RA), and both neovascularization and inflammation also contribute to the pathogenesis of osteoarthritis (OA) (Scanzello et al., 2008; Sokolove and Lepus, 2013). Fibrin deposition is one of the most consistent features of RA in humans and experimental animal models of arthritic disease (Flick et al., 2007), and invasive granulation tissue is present in RA and advanced OA joints (Furuzawa-Carballeda et al., 2008). We have previously shown that synovial tissue of patients with RA and OA harbor EPC and MSC demonstrating the *in vivo* presence of crucial building blocks for postnatal vasculogenesis in an inflammatory microenvironment (Rüger et al., 2004; Giurea et al., 2006). In this study we aimed to provide a platform to investigate the interplay between neovascularization and inflammation. We hypothesized that small pieces of tissues infiltrated with inflammatory cells might be capable to generate neo-vessels when cultured in a biologically relevant 3D environment, even in the absence of exogenously added pro-angiogenic growth factors. We argued that the explant culture would provide a model that integrates complex cellular interactions and paracrine signals involved in pathological neovascularization. Therefore we established a 3D fibrin matrix system for the culture of inflamed synovial tissue fragments of RA and OA patients as exploratory *in vitro* tool reflecting the complexity of remodeling *in vivo*, including both vascular compartment and perivascular inflammatory environment and their cell-cell interactions and paracrine signaling. In order to specifically address the contribution of perivascular cells to neo-vessel formation, the interaction of MSC with peripheral blood mononuclear cells (PBMC) was studied in 3D fibrin matrices. Outgrowth cells/structures, cellular re-arrangement and architectural re-structuring within the first 2 weeks of culture were characterized within topographically intact whole 3D fibrin matrices by a specially adapted 3D-culture immunofluorescence method and confocal laser-scanning microscopy (CLSM), and the release of relevant cytokines, e.g., G-CSF, VEGF, IL-6, and interleukin (IL)-10, during explant- and co-cultures was analyzed.

## Materials and methods

### Ethics statement

The local ethics committee at the Medical University of Vienna approved the use of human synovial tissue (EK 791/2008 and EK1192/2015), human bone marrow MSC (EK1193/2015) and human PBMC (EK1168/2015) in order to perform this study. All donors provided written informed consent.

### Synovial tissue, PBMC, and MSC

Synovial tissues of RA and OA patients were obtained at the time of surgery for arthroplasty or synovectomy. Fresh tissue samples were used for 3D cultures and an adjacent piece was fixed in 4.5% buffered formalin and embedded in paraffin for immunohistochemistry and immunohistochemical double labeling. PBMC were isolated from leukocyte-reduction chambers, a waste product obtained during platelet apheresis, from healthy donors by density grade centrifugation. MSC were isolated from bone marrow and bone fragments obtained during hip-replacement surgery and expanded in complete αMEM medium (Invitrogen, Carlsbad, CA) containing 10% fetal bovine serum (GE Healthcare Life Sciences, Marlborough, MA), 100 U/ml penicillin, 100 μg/ml streptomycin and 250 ng/ml amphotericin B (Sigma, St. Louis, MO) at 37°C (20% O_2_ and 5% CO_2_ humidified atmosphere). MSC were characterized by flow cytometry analyses using CD90FITC (Stem Cell Technologies, Cologne, Germany), CD73PE (BD, Heidelberg, Germany), CD105FITC (BD), CD31PE (Biolegend, San Diego, CA), CD34PE (BD), CD45FITC (BD), and CD14PE (BD) antibodies and a FACS Canto II^TM^ instrument (BD, San Jose, CA). Cells expressed typical MSC markers, CD90, CD73, CD105, lacked expression of CD31, CD34, CD45, and CD14, and could be differentiated into adipocytes, chondrocytes and osteoblasts. For 3D culture experiments passage two to four MSC were used.

### 3D synovial tissue explant culture system

Freshly excised synovial tissue samples (2–4 mm diameter fragments) from 10 OA patients and 8 RA patients were embedded in fibrin scaffolds in individual wells of 24-well culture plates (Corning, Corning, NY). Fibrin matrices were prepared as described previously with minor modifications (Rüger et al., 2008). In brief, human fibrinogen (2 mg/ml; Calbiochem, Darmstadt, Germany) was dissolved in PBS supplemented with 200 U/ml aprotinin (Gerot Pharmaceutica, Vienna, Austria) to prevent fibrinolysis. Human plasma thrombin (0.6 U/ml, Sigma) was added to the fibrinogen solution and gel formation occurred by incubation at 37°C for 30 min. The tissue fragments were cultured using complete M199 medium (Invitrogen) containing 10% fetal bovine serum (GE Healthcare Life Sciences), 100 U/ml penicillin, 100 μg/ml streptomycin and 250 ng/ml amphotericin B (Sigma) without additional growth factors for up to 4 weeks. The culture medium was changed every 3–4 days. The egress of cells into the fibrin matrix, cell growth and structural re-organization including vascular tube formation were monitored using a phase contrast microscope (Olympus IMT-2, Tokyo, Japan) and documented using a digital camera (Olympus DP50). The length of vascular sprouts was assessed in tissue fragments of four OA and RA patients, respectively, after 3 weeks of culture using the cell^*^ Imaging Software (Olympus).

### Co-culture of PBMC with stromal cells in 3D fibrin matrices

3D co-cultures of PBMC with MSC (*n* = 6) were performed in 12-well plates (Corning). Cells were embedded in fibrin matrices in a ratio 1:100 (MSC:PBMC) using 2.5 × 10^6^ PBMC/cm^2^ and cultured for up to 2 weeks in complete αMEM medium (Invitrogen) containing 10% fetal bovine serum (GE Healthcare Life Sciences). Fibrin matrices were prepared as described above, but without addition of aprotinin. Control experiments were performed culturing PBMC separated from MSC by a 0.4 μm transwell insert (Corning), PBMC without support of stromal cells and MSC alone. To investigate the effect of paracrine inflammatory signals on stromal cells in the absence of immune cells, MSC (*n* = 4) were embedded in fibrin matrices in 24-well plates (Corning) at 2.5 × 10^4^ cells/cm^2^ and cultured for 6 days in complete αMEM medium (Invitrogen) containing 10% fetal bovine serum (GE Healthcare Life Sciences) supplemented with 5 ng/ml tumor necrosis factor (TNF)α (PeproTech, Rocky Hill, NJ) and 10 ng/ml interferon (IFN)γ (PeproTech). Control experiments were performed in complete αMEM medium without cytokine supplementation. Cultures were maintained at 37°C (20% O_2_ and 5% CO_2_ humidified atmosphere), and medium was changed every 3 days. Cellular re-arrangement was monitored using a phase contrast microscope (Olympus) and documented using a digital camera (Olympus).

### Cell tracking

In order to investigate the physical interaction of MSC with PBMC in the 3D matrix, MSC were labeled with Cell Tracker Orange fluorescent probe (Molecular Probes, Thermo Fisher Scientific, MS, USA) and PBMC with Cell Tracker Green fluorescent probe (Molecular Probes) according to the manufacturers protocol. Cells were mixed in a ratio of 1:100 (MSC:PBMC), embedded in fibrin gels and cultured for up to 7 days in high resolution chamber slides (ibidi) using complete αMEM medium. For CLSM, cells were fixed with 4% paraformaldehyde and nuclei stained with DAPI.

### Immunohistochemistry and double labeling

Fibrin gels containing the synovial tissue were fixed in formalin and processed for paraffin embedding. Immunohistochemistry was performed on 5 μm sections using the following mouse monoclonal antibodies, CD45 (1.4 μg/ml, Dako, Glostrup, Denmark), CD34 (2 μg/ml, Immunotech, Marseille, France) and podocalyxin (2 μg/ml, kindly supplied by Prof. Dontscho Kerjaschki, Department of Pathology, Medical University Vienna, Austria), two markers for both endothelial and stem/progenitor cells, endothelial markers CD31 (4.5 μg/ml, Dako) and von Willebrand factor (vWF) (5.8 μg/ml, Dako), CD68 (0.5 μg/ml, clone KP-1, Dako), Collagen type-IV (Col-IV) (3.2 μg/ml, Dako), bcl-2 (3.2 μg/ml, Dako), cleaved caspase-3 (4 μg/ml, Cell Signaling Technology, Danvers, MA) and c-kit (CD117) (1.3 μg/ml, rabbit pAb, Dako) together with the Vectastain ABC kit (Vector, Burlingame, CA). Control experiments were included by omission of primary antibodies. Visualization of antibody binding was achieved by 3-amino-9-ethyl-carbazole (Sigma), followed by counterstaining with Mayer's hemalum. Double labeling of cleaved caspase-3 and bcl-2 was performed using a peroxidase/alkaline phosphatase technique as reported previously (Rüger et al., 2004).

### Confocal laser scanning microscopy of intact 3D cultures

In order to perform CLSM of whole 3D cultures, synovial tissue samples (eight, 12 and 13 tissue fragments from three individual OA patients, respectively) were embedded in fibrin matrices using special imaging chambers for high resolution microscopy (ibidi GmbH, Martinsried, Germany). Neo-vessels and the surrounding cellular and extracellular microenvironment present in the fibrin scaffolds were characterized by immunofluorescence analyses using a modified protocol of a previously published method (Lee et al., 2007). Immunofluorescence analyses of intact 3D PBMC-MSC co-cultures were performed in 12-well plates and stained fibrin gels transferred to ibidi chambers for CLSM. Briefly, fibrin matrices were fixed with 4% paraformaldehyde and incubated with a buffer solution containing 0.1% BSA, 0.2% Triton X-100, 0.05% Tween 20 in PBS followed by a blocking step with 20% normal donkey serum (Jackson Immuno Research, West Grove, PA). Fibrin gels were incubated with anti-human CD31 (mouse IgG_1_, 8 μg/ml, Dako) or anti-human CD34 (mouse IgG_1_, 4 μg/ml, Cell Marque, Rocklin, CA) together with rabbit anti-Col-IV (7.5 μg/ml, Novus Biologicals, Cambridge, UK) for 6 h at room temperature. The cultures were washed with buffer solution and incubated simultaneously with donkey anti-mouse IgG_1_ Alexa Fluor (AF)488 and donkey anti-rabbit AF555 (2.6 μg/ml, Molecular Probes, Life Technologies, Carlsbad, CA) and cell nuclei stained with DAPI. CD34/α-smooth muscle actin (α-SMA) double-labeling was performed using monoclonal α-SMA antibody (mouse IgG_1_, clone HHF35, 1 μg/ml, Sigma) and donkey anti-mouse IgG_1_ AF555 followed by a blocking step using 20% mouse serum (Jackson Immuno Research), and incubation with biotinylated CD34 (mouse IgG_1_, 10 μg/ml, Stem Cell Technologies, Cologne, Germany) followed by Streptavidin AF488 (2.6 μg/ml, Molecular Probes, Life Technologies). For triple labeling, the gels were incubated simultaneously with antibody cocktails CD31/Col-IV, CD31/STRO-1/Col-IV (STRO-1, mouse IgM, 10 μg/ml, R&D Systems, Minneapolis, MN), CD34/STRO-1/Col-IV and CD34/STRO-1/c-kit (c-kit, rabbit pAb, Dako), followed by isotype- and species-specific AF-labeled secondary antibody cocktails (Molecular Probes, Life Technologies). Omission of primary antibodies and the use of isotype-matched non-immune antibodies served as controls. After blocking with 20% mouse serum, the 3D constructs stained with CD31 and Col-IV antibodies were incubated with AF647-mouse anti-CD45 (2.5 μg/ml, Biolegend). The cultures were washed with buffer solution, cell nuclei stained with DAPI, and 3D gels kept in PBS at +4°C until CLSM analyses. All 3D cultures were evaluated using a LSM 700 or LSM 780 confocal laser scanning microscope (Carl Zeiss, Jena, Germany) and the acquired images analyzed with the ZEN image processing and analysis software program (Zeiss).

### Cytokine determination

The levels of G-CSF, VEGF, IL-6, and IL-10 were determined by the Bio-Plex 200 system (Bio-Rad, Vienna, Austria) in cell-free explant culture supernatants derived from three OA patients. In all three cases, samples were taken at times of medium exchange at days 4, 8, 15, and 19. We chose tissues from OA patients whose therapy is usually limited to pain control to avoid possible effects of the potent disease-modifying anti-rheumatic drugs commonly taken by RA patients. To account for intra-tissue variations three separate tissue fragments from each individual patient cultured in separate wells were used. Experiments were performed with each sample in duplicate, and data are expressed as mean values ± SD. The supernatants of 3D MSC-PBMC co-cultures were taken after 24 h, on days 3 and 6; supernatants of the corresponding 3D PBMC- and MSC-monocultures were taken on day 6. G-CSF, VEGF, and IL-6 levels were determined using commercially available ELISA Duoset systems (R&D). In 3D co-cultures where MSC and PBMC were physically separated by a transwell insert, G-CSF and VEGF levels in the supernatants were measured by ELISA (R&D) on day 6. Supernatants of MSC cultured in fibrin gels with and without inflammatory stimulation were taken on days 3 and 6, and VEGF and G-CSF levels analyzed by ELISA (R&D). The assays were performed according to the reference manual, and the samples were measured in technical duplicates. Optical density values were measured at 450 nm on an ELISA plate reader (Victor3 Multilabel plate reader, PerkinElmer).

### Statistics

Statistical analyses were performed using the software package SPSS Statistics for Windows, version 22.0 (SPSS Inc., Chicago, IL). When comparing two groups, data were analyzed by the non-parametric Wilcoxon rank sum test. Data are expressed as means ± SD. Significance was accepted at *p* ≤ 0.05 (^*^), *p* ≤ 0.01 (^**^), and *p* ≤ 0.001 (^***^).

## Results

### Vascular outgrowth from synovial tissue explants is preceded by leukocyte egress

**Figure 1:**
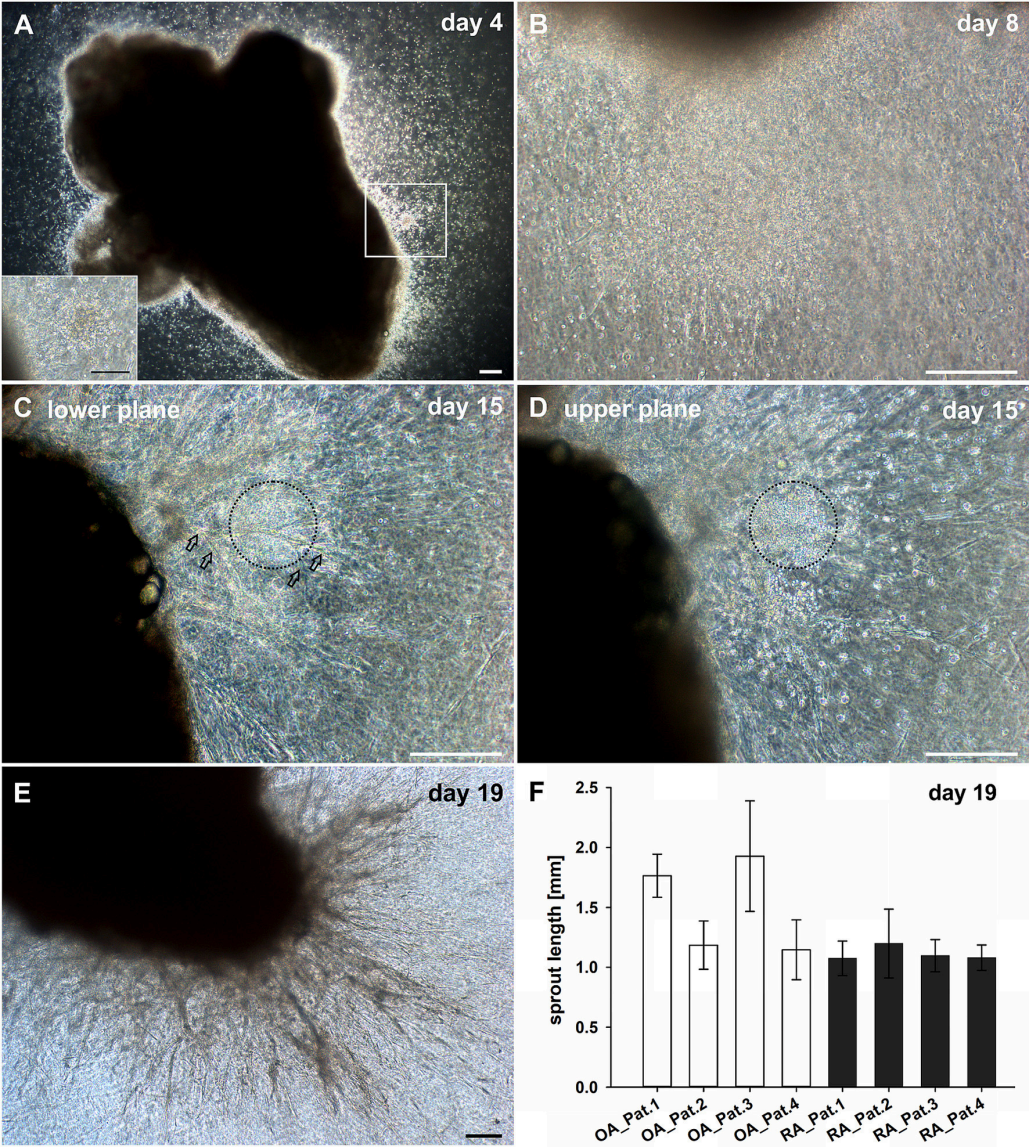
Culture of synovial tissue in fibrin gels leads to the development of vascular structures preceded by egress of inflammatory cells into the 3D matrix. **(A)** Mononuclear cells present at day four around the embedded explant tissue forming a cluster (insert, represents magnified area of white box). **(B)** Dense infiltration of mononuclear cells invading the fibrin gel accompanied by spindle-shaped cells after 1 week of culture. **(C)** Neo-vessels emanating from the synovial tissue after 2 weeks of culture (open arrows). **(D)** Vascular sprouts develop in close proximity to inflammatory cells (black dashed circles). Images **(C,D)** represent two different planes of the same area within the gel (**C**, lower plane; **D**, upper plane). **(E)** A complex vascular network is present within the fibrin gel after 19 days of explant tissue culture. **(A–E)** Sequential phase contrast microscopy images of representative inflammatory OA explant tissue in 3D fibrin gel taken on indicated days. **(F)** Maximum length of vascular sprouts measured in representative explant cultures of four OA and RA patients, respectively. Scale bars, 200 μm.

With the aim to explore the mechanisms of new vessel formation in the context of inflammation under conditions that closely mimic the *in vivo* situation, a 3D explant model preserving the complexity of tissue architecture and environment was established. Intact synovial tissue fragments from patients with RA and OA containing high numbers of CD45^+^ inflammatory cells (Supplemental Figure 1E) were cultured for up to 4 weeks in fibrin matrices in the absence of additional pro-angiogenic growth factors. Dependent on the size of the synovial tissue samples obtained, five to ten tissue fragments from each patient were sandwiched between two fibrin gels in individual wells of the culture plate. We chose fibrin as matrix as it occurs at virtually any site of overt tissue damage and forms a three-dimensional scaffold that provides cell-attachment sites and mechanical support for infiltrating leukocytes as well as invading endothelial and stromal cells. Phase contrast microscopy was a suitable tool to observe the spatial organization of the cellular outgrowth in the initially avascular and translucent fibrin matrix. Noticeable in all samples with considerable inflammation was that in the first days of culture, tissue-derived mononuclear cells migrated into the fibrin matrix (Figure 1A) occasionally forming cell clusters (Figure 1A, insert). Spindle-shaped cells appeared within the first week of cultivation emanating from the explant and radially growing into the 3D fibrin gels infiltrated with the previously invaded mononuclear cells (Figure 1B). Peripheral spindle-shaped cells formed cellular strands that continued to grow outward building cord like structures (Figure 1C). The sprouts developed in different planes within the fibrin gel and were closely associated with mononuclear cell clusters (Figure 1D). After 3 weeks of culture complex vascular structures measuring up to one and two mm of length were detected (Figures 1E,F). There were no apparent differences in vascular outgrowth between RA and inflammatory OA samples. One to three tissue fragments from four RA and three OA patients did not show mononuclear cell egress and the outgrowth was predominantly fibroblastic (Supplemental Figure 2A). In accordance, histological analysis of such cultured tissue fragments revealed only negligible inflammation (data not shown).

### Neo-vessels develop in close physical contact with inflammatory cells

**Figure 2:**
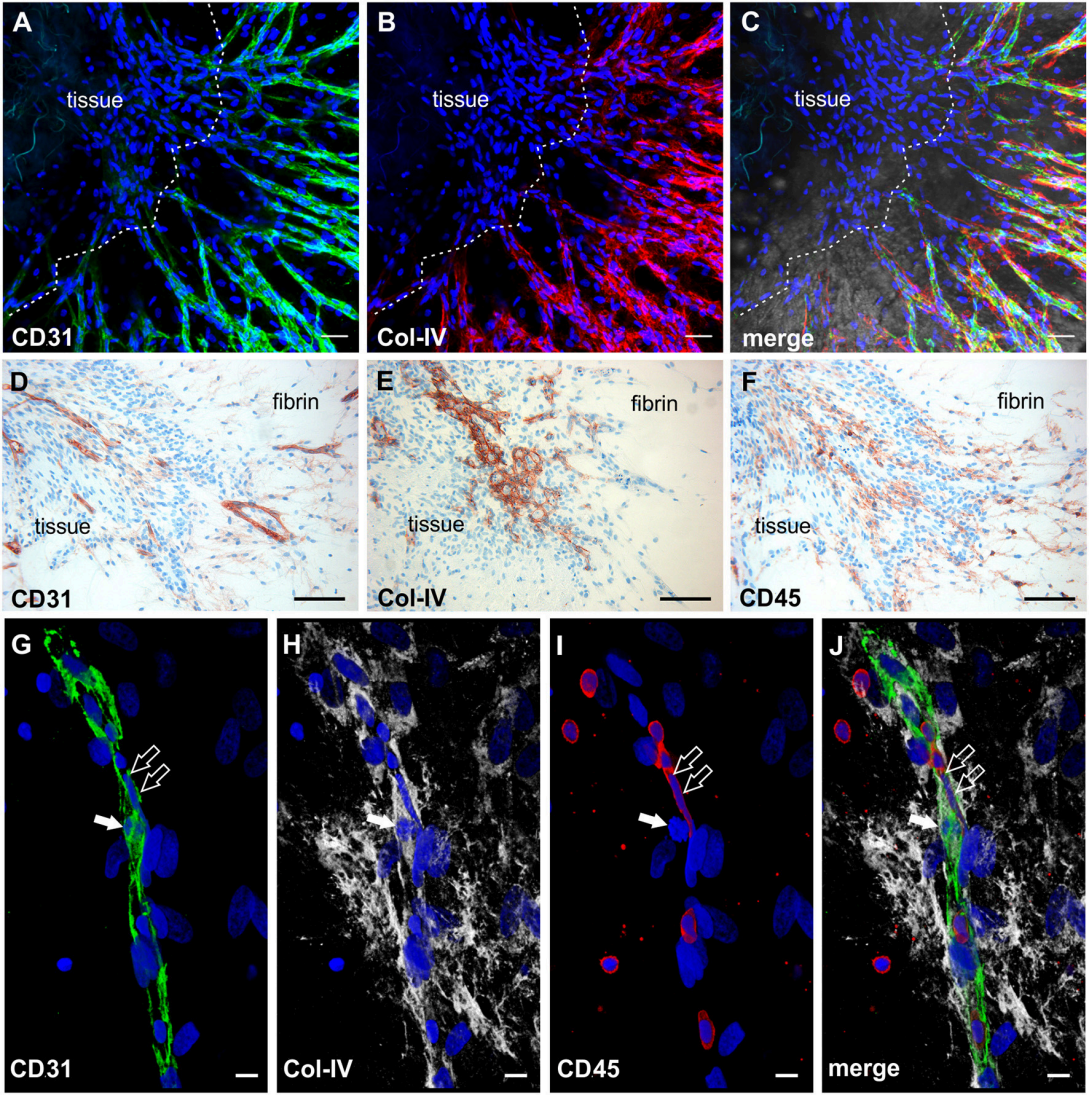
Neo-vessel growth from synovial explants occurs in association with inflammatory cells. Complex vascular network within the fibrin matrix originating from the embedded tissue fragment expressing **(A)** CD31 (green) and showing **(B)** Col-IV positive basement membrane and mural cells (red). **(C)** Merged image of **(A,B)** demonstrating partial co-expression of CD31 and Col-IV. The tissue itself is unstained due to poor penetration of antibodies. **(A–C)** CLSM images of an intact fibrin gel on day 19, collapsed 13 μm-z-stack consisting of 9 consecutive images. Scale bars, 50 μm. **(D)** Cultured tissue fragment showing CD31^+^ vessels that grow in direction of the surrounding fibrin matrix and CD31^+^ vascular structures within the fibrin gel. **(E)** Cultured synovial tissue containing numerous round Col-IV positive clusters. **(F)** Intra-synovial neo-vessels aligned and closely associated with CD45^+^ inflammatory cells. **(D–F)** Immunohistochemistry on consecutive paraffin sections of a day 28 explant culture sample. Scale bars 100 μm. **(G–J)** Immature vascular sprout developing within the fibrin matrix consisting of **(G)** CD31^+^ endothelial cells that are surrounded by **(H)** Col-IV^+^ stromal cells. **(I)** Numerous CD45^+^ leukocytes are integrated in the growing cell cord and surround the vascular sprout. Note the CD45^−^ dividing cell that co-expresses CD31 and Col-IV (arrow) and the elongated nucleus of the CD45^+^ cell (open arrows) with **(J)** partial co-expression of CD31. CLSM images of an intact fibrin gel on day 20, collapsed 15 μm-z-stack consisting of 16 consecutive images. All images are shown with DAPI counterstain in blue. Scale bars, 10 μm.

The phenotypic characterization of vascular structures in fibrin gels was performed by immunohistochemistry and double labeling on paraffin sections, and immunofluorescence staining and CLSM of intact 3D cultures. Neo-vessels present in fibrin gels originating from the embedded tissues consisted of CD31^+^ endothelial cells surrounded by cells expressing Col-IV and a Col-IV positive basement membrane with some CD31^+^ cells co-expressing this ECM protein (Figures 2A–C). The newly formed vascular structures showed positive staining for several other endothelial cell markers including CD34, podocalyxin and vWF (Supplemental Figures 1A–C), and were surrounded by α-SMA^+^ mural cells (Supplemental Figure 3). Among the outgrowth cells were also cells expressing CD68, most of them not showing the typical appearance of monocytes/macrophages indicating a possible non-myeloid origin (Gottfried et al., 2008). Interestingly, the architecture of the embedded synovial tissue samples appeared intact even after 4 weeks of culture in the 3D fibrin matrix (Figures 2D–F and Supplemental Figure 1F). Immunohistochemistry on sections of cultured tissue fragments could be performed successfully and showed that vessel growth—as assessed by CD31 staining—was exclusively directed toward the fibrin matrix (Figure 2D). The spatial organization of CD45^+^ inflammatory cells still present in the cultured tissue demonstrated a similar orientation where leukocytes were aligned and closely associated with the intra-synovial vascular sprouts (Figure 2F). Neo-vessels were surrounded by cells/clusters strongly expressing Col-IV (Figure 2E). There was a noticeable decrease of CD45^+^ cells in the cultured tissues (Supplemental Figure 1F) when compared to pre-culture control sections of an adjacent tissue fragment (Supplemental Figure 1E). This was not entirely surprising due to the considerable egress of mononuclear cells from the tissues into the fibrin gel during culture which obviously led to a reduction of inflammatory cells within the explant fragments. The close physical association of immune cells with developing neo-vessels was also found in vascular sprouts newly formed *within* the fibrin matrix as demonstrated by triple-immunofluorescence staining of intact 3D cultures for CD45, CD31, and Col-IV. Here, CD45^+^ cells were found intimately lodged alongside immature proliferating CD31^+^ endothelial cells that co-expressed Col-IV (Figures 2G–J).

### Cell clusters formed by emigrated mononuclear cells are an origin of neo-vessels

**Figure 3:**
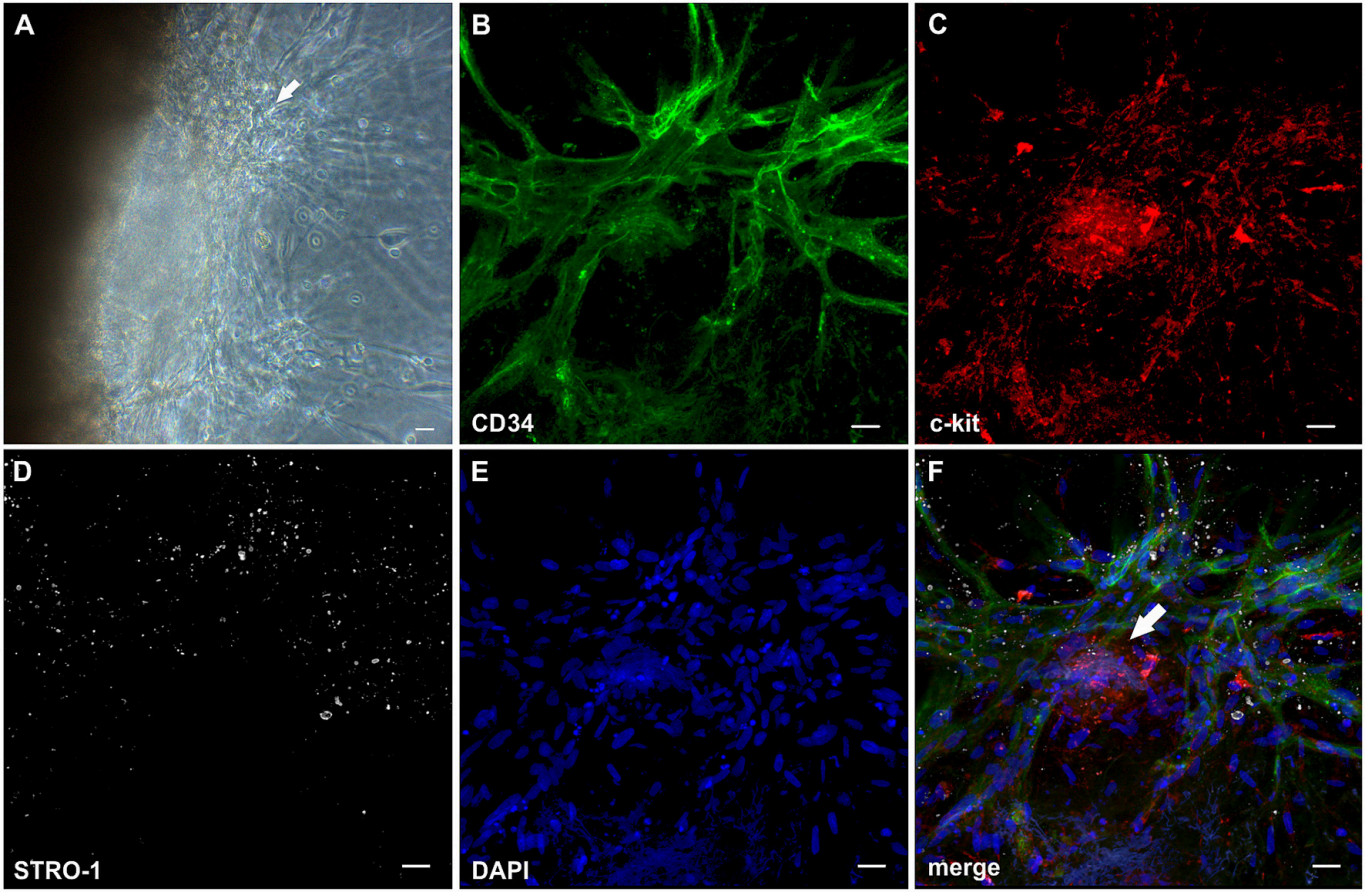
Cell clusters within fibrin gels are an origin of neo-vessels formed by vasculogenesis. **(A)** Phase contrast microscopy image of a representative inflamed synovial tissue sample in 3D fibrin matrix on day 10 of culture, showing a cell cluster (arrow) external from the embedded tissue fragment in the fibrin gel with outgrowing elongated structures. The image was taken before IF staining. **(B–F)** CLSM images (collapsed 32 μm-z-stack consisting of 20 consecutive images) showing the same cluster (arrow in **F**) depicted in **(A)**. Both cluster and neo-vessels co-express **(B)** CD34 and **(C)** c-kit. **(D)** MSC marker STRO-1. **(E)** DAPI stain. **(F)** Merge. Scale bars, 20 μm.

Mononuclear cells found in the fibrin gels after egress from synovial explant fragments formed cell clusters external from the tissue within the 3D matrix (Figure 1A, insert). From these clusters vascular sprouts emanated (Figure 3A) resembling developmental vasculogenesis. Considering the phenotypic similarities to previously described vasculogenic clusters in 3D fibrin cultures of unselected bone marrow mononuclear cells (Rüger et al., 2008), we investigated the progenitor marker profile of these clusters using the stem cell markers CD34, c-kit and STRO-1. While clusters and neo-vessels expressed CD34 (Figures 3B,F) and c-kit (Figures 3C,F), the mesenchymal stem cell marker STRO-1 was detected alongside newly forming vessel sprouts in a predominantly dotty staining pattern (Figures 3D,F), consistent with the perivascular STRO-1 deposition found previously in RA and OA synovial tissue (Rüger et al., 2004; Giurea et al., 2006). Similar clusters with apparent vascular outgrowth were also found *within* the embedded tissue samples after 9 days of culture, i.e., shortly before or around the time of the first appearance of vascular tubes in the fibrin matrix. Here, the neo-vessels emanating from Col-IV positive intra-tissue cell clusters expressed CD34, CD31, vWF, c-kit and bcl-2 surrounding caspase 3 expressing apoptotic cells (Supplemental Figure 4). Numerous cells with condensed nuclei were also present in the immature sprouts originating from clusters formed externally from the synovial tissue (Figure 3E).

### Lumen formation is associated with apoptosis of central vascular cord cells

**Figure 4:**
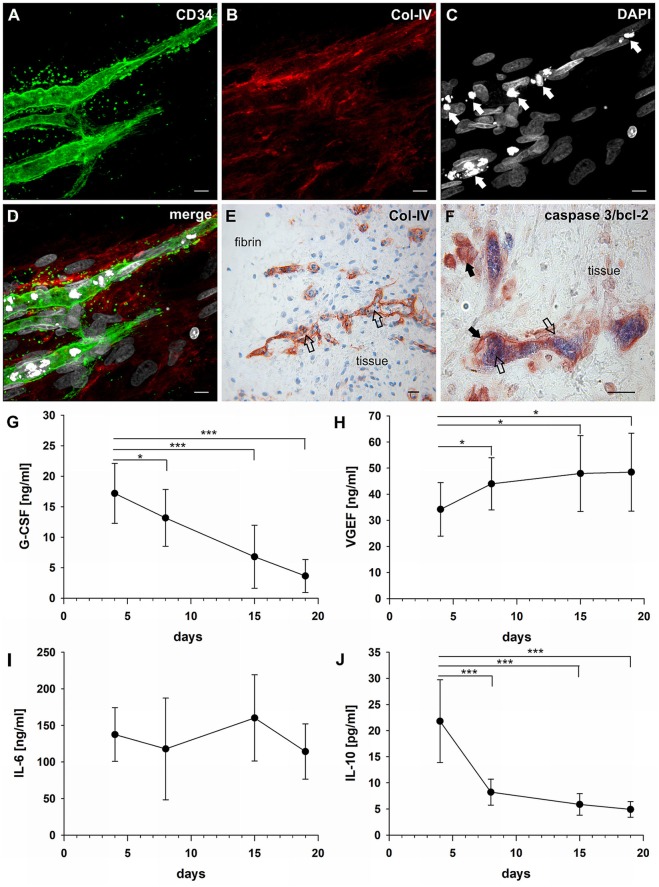
Vascular lumen formation is associated with apoptosis. Immature vascular sprout within the fibrin matrix expressing **(A)** CD34 and surrounded by **(B)** Col-IV^+^ stromal/mural cells. **(C)** DAPI stain showing that the core of the developing vascular sprout contains several cells with condensed nuclei (arrows). **(D)** Merge. **(A–D)** CLSM images of intact fibrin gel culture of OA synovial tissue on day 21, collapsed 13 μm-z-stack consisting of 17 consecutive images. Scale bars, 10 μm. **(E)** Neo-vessels of cultured synovial tissue surrounded by Col-IV positive basement membrane and mural cells showing intraluminal cells with condensed nuclei (apoptotic cells) *within* and *outside* of the embedded tissue fragment (open arrows). Note the regular nuclear morphology of the extravascular cells within the tissue after extended explant culture. **(F)** Caspase-3^+^ intraluminal apoptotic cells (blue, open arrows) *within* the synovial tissue surrounded by bcl-2^+^ endothelial cells (arrows). **(E)** Immunohistochemistry and **(F)** peroxidase/alkaline phosphatase double-labeling on paraffin sections of RA explant tissue cultured for 28 days. Scale bars, 20 μm. Kinetics of cytokine secretion during inflammatory neo-vessel development. Determination of **(G)** G-CSF, **(H)** VEGF, **(I)** IL-6, and **(J)** IL-10 using Bio-Plex 200 system in cell-free explant culture supernatants from three patients with inflammatory OA (*n* = 9) taken at days 4, 8, 15, and 19, respectively. Experiments were performed with each sample in duplicate, and data are expressed as mean values ± SD. ^*^p ≤ 0.05, ^***^p ≤ 0.001.

The establishment of a vascular lumen is an important developmental step concerning the functionality of newly formed blood vessels. In our model—a static system in the absence of a blood circulation—lumens appeared to be formed in association with cell apoptosis. Apoptotic cells were present in the core of vascular cords developing in the fibrin matrix (external from the embedded tissue) (Figures 4A–D) showing expression of CD31 (Supplemental Figure 5 and Supplemental Video 1), and also within the cultured synovial tissue where they were localized exclusively to the intraluminal space of vessels (Figure 4E). The intraluminal cells with typical condensed nuclei expressed caspase 3 and were surrounded by bcl-2^+^ endothelial cells reflecting the interdependent mechanisms of co-ordinated *construction* and *deconstruction* during a morphogenetic process (Figure 4F).

### G-CSF, VEGF, and IL-6 are secreted during inflammatory neo-vessel development

In order to investigate the paracrine signature during neo-vessel formation by synovial explant tissues we analyzed the time-dependent release of relevant cytokines/growth factors typically involved in inflammation and neovascularization. The secretion of G-CSF, VEGF, IL-6, and IL-10 were determined in cell-free supernatants of 3D-cultured synovial explants from three individual osteoarthritis patients (Figures 4G–J). G-CSF levels were extremely high on day four of culture (17 ng/ml ± 5) and gradually declined over time, but were still significantly above normal serum levels on day 19 (4 ng/ml ± 3) (Figure 4G). The time-dependent secretion of VEGF showed an inverse correlation with the corresponding G-CSF release. High VEGF values on day four (34 ng/ml ± 10) gradually increased even further during culture (day 19: 48 ng/ml ± 15) (Figure 4H). The increase of VEGF coincided with the progressive neo-vessel growth in the 3D gels. IL-6 secretion of inflamed synovial explant tissues was high during the whole culture period with levels above 100 ng/ml, but without statistically significant change over time (Figure 4I). IL-10 levels were low on day four (22 pg/ml ± 8), sharply declined further during the first week of culture and remained low (Figure 4J). When we analyzed the cytokine secretion in one of the tissue fragments showing fibroblastic outgrowth, considerably lower G-CSF, VEGF and IL-6 levels were measured compared to inflammatory OA samples (Supplemental Figures 2B–D).

### Co-cultures of MSC and PBMC form cell clusters and secrete VEGF, G-CSF, and IL-6

**Figure 5:**
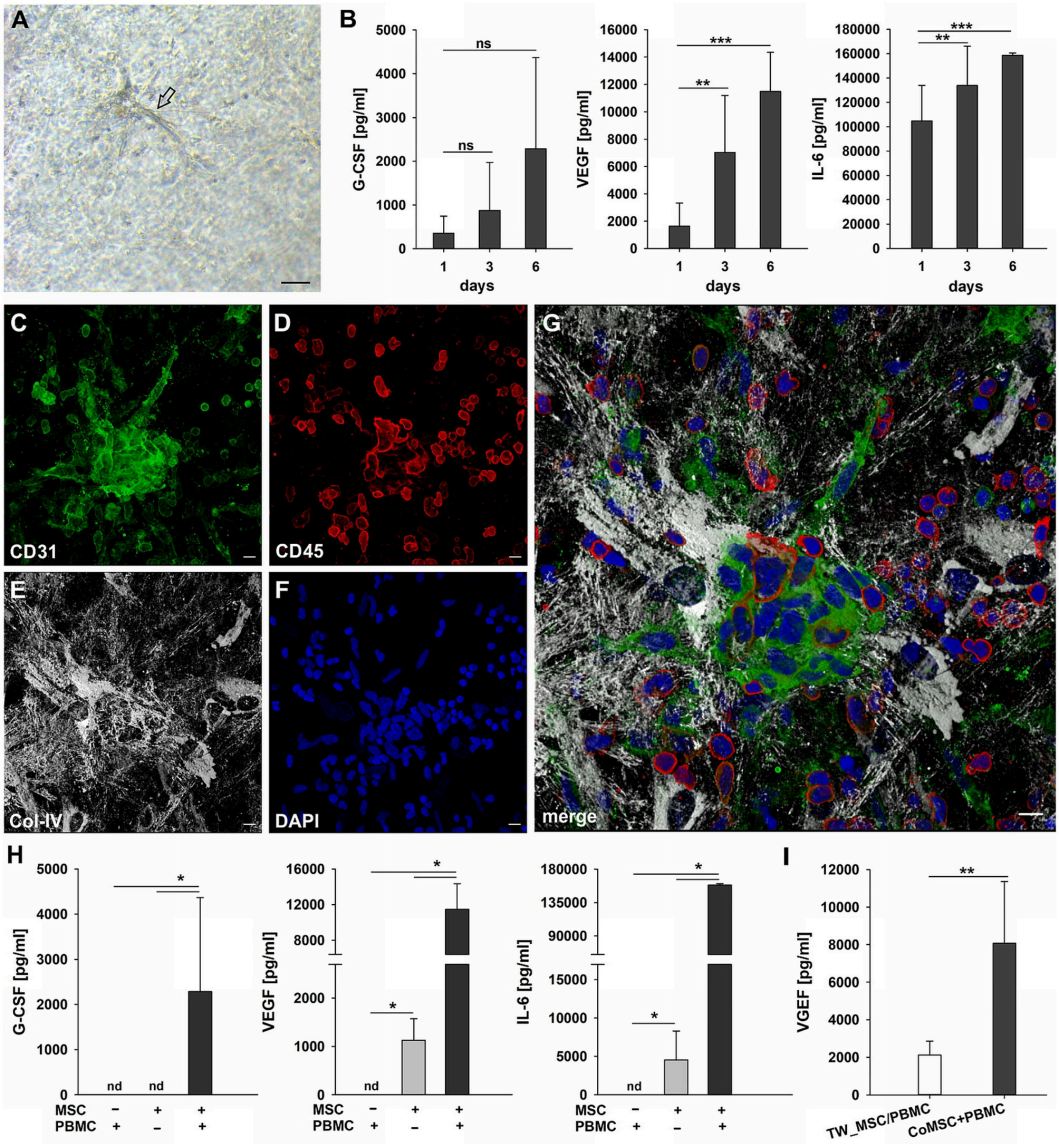
Cluster formation, paracrine signaling and emergence of endothelial cells in 3D MSC-PBMC co-culture. **(A)** Phase contrast microscopy image of representative 3D fibrin co-culture of MSC with PBMC on day 6, demonstrating vascular sprouts (arrow). Scale bar, 50 μm. **(B)** Determination of G-CSF, VEGF and IL-6 by ELISA in cell-free supernatants of 3D MSC-PBMC co-cultures (*n* = 6) after 24 h, on day 3 and on day 6, respectively. The data are expressed as mean values ± SD. ^**^p ≤ 0.01. ^***^p ≤ 0.001. ns not significant. **(C–G)** Representative CLSM images of MSC-PBMC co-culture in 3D fibrin matrix on day 6 showing organized structures consisting of **(C)** CD31^+^ cell clusters with **(D)** partial co-expression of CD45. Note the numerous CD45^+^ round leukocytes surrounding the cell cluster. CD31^+^/CD45^−^ outgrowth cells with EC morphology emerge from the cluster. The cluster is embedded in a meshwork of **(E)** Col-IV positive stromal cells and matrix. **(G)** Merge. **(F)** DAPI stain. Collapsed 16 μm z-stack consisting of 33 consecutive images. Scale bars, 10 μm. **(H)** Determination of G-CSF, VEGF and IL-6 by ELISA in cell-free supernatants of 3D PBMC mono-cultures (*n* = 4), MSC mono-cultures (*n* = 4) and corresponding MSC-PBMC co-cultures on day 6. **(I)** VEGF levels in cell-free supernatants of 3D co-cultures where MSC were physically separated from PBMC by a transwell insert with 0.4 μm pore size and corresponding 3D MSC-PBMC co-cultures on day 6 (*n* = 6). The data are expressed as mean values ± SD. nd = not detected. ^*^p ≤ 0.05. ^**^p ≤ 0.01.

MSC as progenitor cells of the connective tissue are found in almost all organs throughout the body residing mostly close to blood vessels (Crisan et al., 2008), where they play an essential role in regenerative processes through co-operation with inflammatory cells. To simulate the cross-talk between MSC and immune cells in the perivascular space and its contribution to vascular remodeling in a pro-inflammatory environment we co-cultured bone marrow- and bone-derived MSC and PBMC in fibrin matrices for up to 14 days. Co-cultures were performed using α-MEM medium containing 10% FBS, but without additional growth factors. In a self-organization process, MSC co-cultured with PBMC formed clusters in the fibrin matrix from which cellular strands emanated (Figure 5A). The formation of cell clusters and their sprouting was detected from day four onwards and could be observed throughout the culture period (Figure 5, Supplemental Figure 6). The elongated cells originating from the clusters displayed an endothelial phenotype expressing CD31 (Figure 5C) and lacking CD45 staining (Figure 5D), and were surrounded by Col-IV^+^ cells (Figure 5E). No fibrinolysis was evident in co-cultures. Cluster cells expressed CD31, showed close contact with CD45^+^ leukocytes partly co-expressing CD31 (Figure 5G) and were enwrapped in a network of stromal cells producing a collagen IV^+^ vault-like scaffold (Figures 5E,G, Supplemental Figures 6C,E, Supplemental Videos 2, 3). MSC did not form clusters in fibrin gels in the absence of PBMC, and no vascular-like structures were detected (data not shown). Based on the first appearance of cell clusters and sprouting on day four, we investigated the production of VEGF, G-CSF and IL-6 by co-cultures within the first week, i.e., after 24 h, on day 3 and on day 6. Secretion of these three cytokines progressively increased over the culture period (Figure 5B), and large amounts of these cytokines were found in the supernatants of co-cultured cells on day 6 (G-CSF, 2285 pg/ml ± 2084; VEGF, 11485 pg/ml ± 2865; IL-6, 158713 pg/ml ± 1921). No cytokine secretion was detected in PBMC cultures without stromal support (Figure 5H), and the gels demonstrated extensive fibrinolysis (data not shown). In the absence of PBMC, MSC did not release G-CSF, whereas detectable levels of VEGF and IL-6 were measured in the supernatants of MSC mono-cultures, albeit significantly lower compared to MSC co-cultured with PBMC (MSC only: VEGF, 1130 pg/ml ± 443; IL-6, 4528 pg/ml ± 3769). VEGF secretion was significantly lower in co-cultures where MSC-PBMC contact was prevented by a transwell insert (0.4 μm pore size) (TW-MSC/PBMC: 2118 pg/ml ± 741; coMSC/PBMC: 8075 pg/ml ± 3290) (Figure 5I). Contact-inhibition also decreased G-CSF secretion in co-cultures, however, the difference did not reach significance level (Supplemental Figure 7).

### MSC respond to paracrine inflammatory signals in 3D fibrin matrices by development of filopodia-like cell protrusions and increased VEGF production

**Figure 6:**
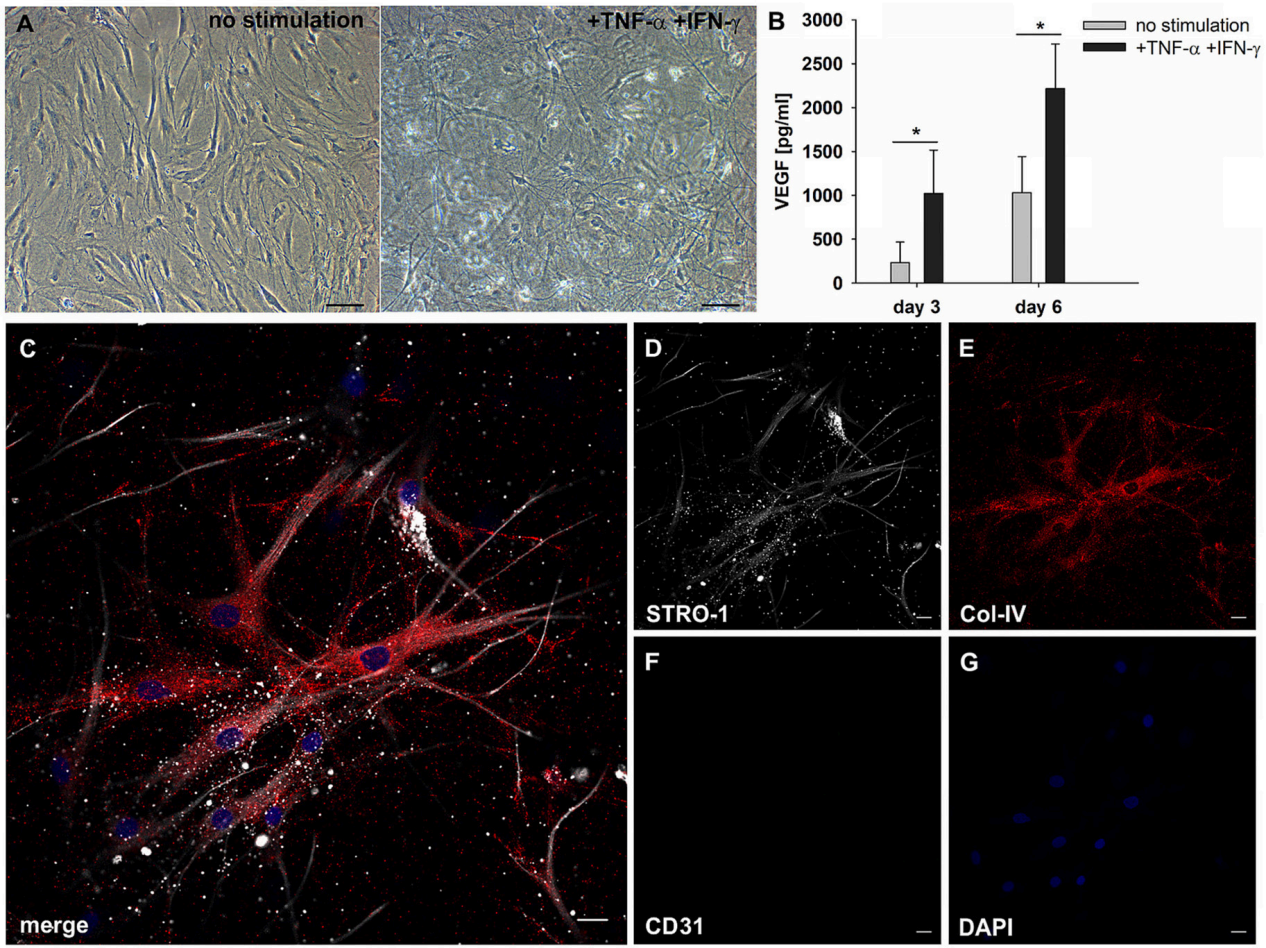
MSC respond to inflammatory signals and develop filopodia. **(A)** Representative phase contrast images of bone marrow-derived MSC cultured in 3D fibrin matrices for 6 days. Unstimulated MSC with mainly spindle-shaped morphology, and TNFα/IFNγ-stimulated MSC showing multiple long cell protrusions resembling filopodia. Scale bars, 100 μm. **(B)** VEGF secretion by unstimulated and stimulated MSC on day 3 and day 6 ^*^p ≤ 0.05. **(C–G)** Representative CLSM images of MSC in 3D fibrin matrix with the addition of TNFα and IFNγ showing **(D)** STRO-1 expression in filopodia. **(E)** MSC produce Col-IV and **(F)** do not express CD31. **(C)** Merge. **(G)** DAPI stain. Scale bars, 20 μm.

MSC are responsive to pro-inflammatory cytokines, and cytokines produced during an immune response, e.g., TNFα and IFNγ, affect the secretion of pro-angiogenic mediators by MSC. MSC were embedded in 3D fibrin matrices, stimulated with TNFα and IFNγ and the release of VEGF and G-CSF was measured in cell-free supernatants by ELISA. Without stimulation MSC released 231 pg/ml ± 234 VEGF on day 3, and the levels increased to 1,021 pg/ml ± 496 after 6 days of culture. VEGF secretion was significantly upregulated by cytokine stimulation (day 3: 1,029 pg/ml ± 413; day 6: 2,216 pg/ml ± 510) (Figure 6B). No G-CSF was detected in the supernatants of either unstimulated or cytokine-stimulated MSC cultures (data not shown). Stimulated MSC did not generate cell clusters, however, the pro-inflammatory microenvironment led to dramatic morphological changes of MSC characterized by the formation of numerous long cell protrusions resembling filopodia (Figures 6A,C). STRO-1 was localized to filopodia showing a predominantly granular staining pattern (Figure 6D). MSC produced collagen IV (Figure 6E), and did not express CD31 (Figure 6F).

### Cellular sprouts emerging from co-culture-derived clusters display a mixed endothelial/mesenchymal phenotype

The notion that MSC can differentiate into endothelial cells is still under debate (Oswald et al., 2004; Corotchi et al., 2013). STRO-1 is an accepted and widely used mesenchymal stem cells marker, and has been described more recently as endothelial antigen (Ning et al., 2011).

**Figure 7:**
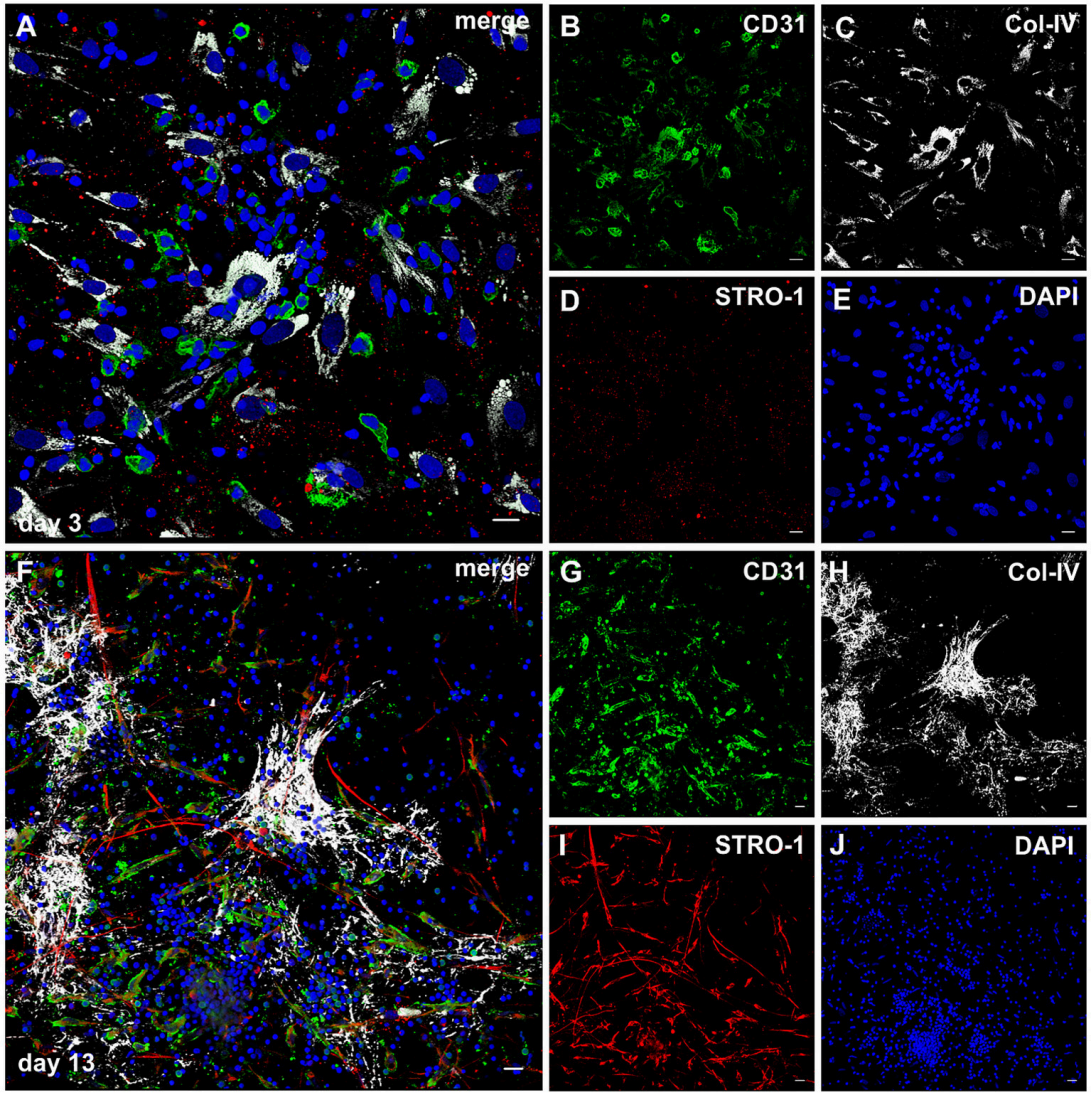
STRO-1 expression during MSC-PBMC co-culture in 3D fibrin gels. **(A)** MSC-PBMC co-culture on day 3 showing close physical contact of mononuclear cells with Col-IV^+^ STRO-1^+^ MSC. Some mononuclear cells are CD31^+^. **(C)** MSC express Col-IV. **(D)** STRO-1 in MSC shows a granular staining pattern. **(E)** DAPI stain. **(F)** 3D MSC-PBMC co-culture on day 13 demonstrating elongated cells that co-express **(G)** CD31 and **(I)** STRO-1. CD31^+^STRO-1^+^ cells are associated with **(H)** Col-IV positive cell clusters. Note that round CD31^+^ cells are STRO-1^−^. STRO-1^+^CD31^−^ MSC show long cell protrusions. **(J)** DAPI stain. **(A–J)** Representative CLSM images of MSC-PBMC co-cultures within 3D fibrin matrices. Scale bars, 20 μm.

Considering the fact that the pro-angiogenic milieu in co-cultures is dependent on MSC-PBMC contact, and given the plasticity of MSC in response to cues from the microenvironment, we investigated the expression of STRO-1 in the fibrin based 3D co-cultures of MSC and PBMC. When structurally organized cell clusters surrounded by a Col-IV scaffold had formed (Figures 7F–J), elongated cells with EC morphology and mixed endothelial/mesenchymal phenotype expressing CD31 and STRO-1 emanated from these clusters (Figures 7G,I, Supplemental Video 4). These hybrid-type cells were absent at day three (Figures 7A–E). CD31^+^ cells with round morphology did not express STRO-1. In control 3D mono-cultures, a minority of MSC expressed STRO-1; in PBMC mono-cultures, STRO-1 was not detected at all (data not shown).

### MSC and leukocytes establish close cell-cell contacts in 3D fibrin matrices

**Figure 8:**
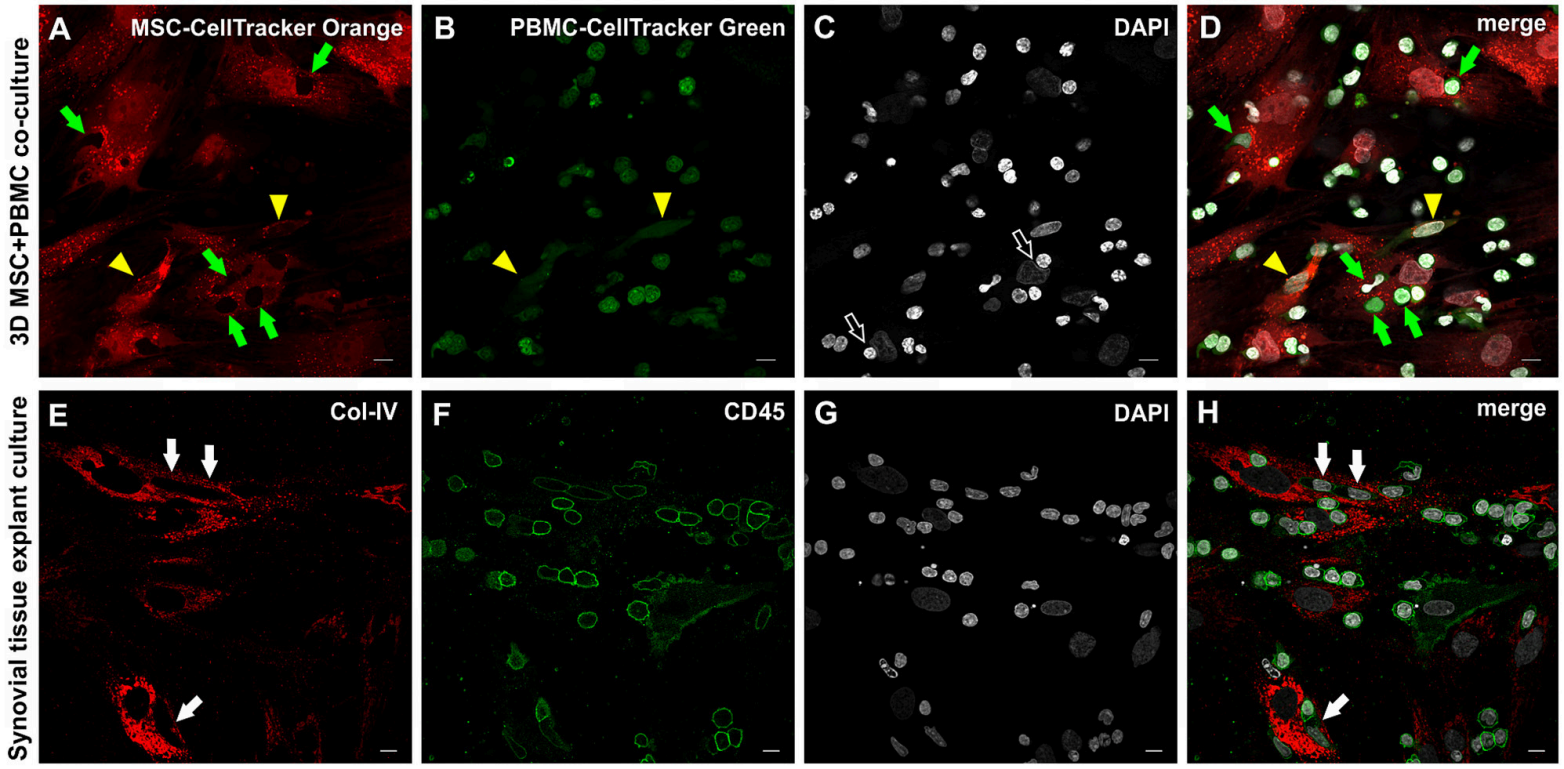
Close physical contact between immune cells and stromal cells in 3D MSC-PBMC co-culture and 3D synovial tissue explant culture. **(A)** MSC labeled with Cell Tracker Orange fluorescent probe and **(B)** PBMC labeled with Cell Tracker Green fluorescent probe showing **(D)** close contact of both cell types with each other (merged image), where leukocytes are in close contact with the cytoplasm of the MSC (green arrows). Note the corresponding empty spaces in the cytoplasm of MSC in **(A)**. Partial co-staining of both dyes is evident in two cells demonstrating an EC-like nucleus (yellow arrowheads in **A**,**B**,**D**). **(C)** DAPI stain showing that some nuclei of stromal cells are indented by round nuclei of PBMC (open arrows). **(A–D)** Representative CLSM images of bone marrow-MSC cultured with PBMC in 3D fibrin gels on day 4. Scale bars, 10 μm. Cellular outgrowth from synovial explant tissue cultured in 3D fibrin gels showing **(E)** Col-IV^+^ stromal cells closely associated with **(F)** CD45^+^ leukocytes. **(H)** Merged image demonstrating the close physical interaction between both cell types. Note that cells with lower CD45 expression in close contact with stromal cells have an elongated EC-like nucleus (white arrows). **(G)** DAPI stain. **(E–H)** CLSM images of intact fibrin gel culture of OA synovial tissue on day 20. Scale bars, 10 μm.

In order to examine the MSC interaction/communication with immune cells in this pro-angiogenic environment, we performed cell tracking experiments. PBMC were labeled with CellTracker Green dye, and co-cultured in 3D fibrin gels for 4–7 days together with CellTracker Orange dye-labeled MSC. PBMC (green, Figure 8B) were detected in intimate contact with MSC (red, Figure 8D) clearly demonstrated by the empty spaces in labeled MSC (Figure 8A) and the partly deformed nuclei of the MSC (Figure 8C) due to the close contact with immune cells. In few cells with elongated nuclei both dyes appeared to be present (Figure 8D, yellow arrowheads). In both, the explant and the co-culture model, MSC-PBMC contact coincided with weak CD45 expression (Figures 8E–H and Supplemental Figures 8A–E). Triple immunofluorescence staining (CD31/CD45/Col-IV) and CLSM of 3D co-cultures revealed the close spatial relationship between cells of the stromal and hematopoietic lineages (Supplemental Figures 8F–J). Cells co-expressing CD31/CD45 showed variations in the expression level of either marker with some cells exclusively expressing CD31. CD31 expression in the absence of CD45 was associated with nuclear elongation possibly indicating differentiation toward the endothelial lineage.

## Discussion

The regeneration of damaged tissues is usually initiated by an inflammatory response that eventually is down-regulated and associated with physiological neovascularization in order to re-establish normal tissue function. If this process is not properly organized, however, it results in a persistent cycle of inflammation and repetitive tissue damage with concomitant excessive neo-vessel formation as it is found in various chronic inflammatory diseases. The cross-talk between both processes—inflammation and neovascularization—occurs in a specific perivascular microenvironment, where key molecular players of the coagulation cascade like fibrinogen/fibrin combined with a complex network of chemical signals and cell interactions initiate and sustain the process (Davalos and Akassoglou, 2012). In this study we have established a contextual *in vitro* model for neovascularization in an inflammatory environment by embedding freshly excised inflamed synovial tissue fragments in 3D fibrin matrices using human fibrinogen at physiological concentrations for the fabrication of malleable translucent scaffolds. This enabled the observation of vascular morphogenesis and cellular interactions, and involved paracrine signaling in a complex inflammatory microenvironment close to the *in vivo* setting (albeit lacking perfusion of the preexisting vessels). The contribution of perivascular niche cell interactions to neo-vessel formation during an inflammatory process was simulated in a complexity-reduced system by co-culturing MSC with freshly isolated PBMC in 3D fibrin matrices. This initially EC-free co-culture set-up provided evidence that *de novo* vessel development can be promoted by the formation of mixed cell clusters with vasculogenic potential and concomitant secretion of pro-angiogenic cytokines.

The 3D explant culture model represents a straightforward method for closely recapitulating complex *in vivo* regenerative processes. The embedded tissue samples remained viable even after extended culture in fibrin scaffolds demonstrating that the diffusion of nutrients was sufficient, not only to support their survival, but also to promote intra-tissue remodeling in this 3D microenvironment. Originating from the embedded synovial tissue, inflammatory mononuclear cells and spindle-shaped cells migrated into the initially acellular 3D fibrin matrices to form a radially emanating network of increasing density leading to the development of complex organized neo-vessels within 3–4 weeks. The core of the newly formed vascular structures consisted of endothelial cells as indicated by expression of the common endothelial markers CD31, CD34, vWF, and podocalyxin, and was surrounded by a Col-IV^+^ basement membrane and by cells also expressing Col-IV. Neo-vessels emerged directly from the tissue fragments developing from intra-synovial cell clusters expressing Col-IV and growing outward into the fibrin gels, and in addition, were created via cell clusters formed by emigrated mononuclear cells found in the fibrin matrix surrounding the explant tissue. CD45^+^ cells were consistently seen integrated within and around developing vascular sprouts. Similar clusters with vasculogenic potential were formed when PBMC where cultured in fibrin gels together with MSC. Cells with endothelial morphology and phenotype (CD31^+^CD45^−^) emerged from CD31^+^ cell clusters showing close physical contact with CD45^+^ leukocytes and a surrounding Col-IV^+^ scaffold demonstrating that the co-operation of cells of the hematopoietic and mesenchymal lineages initiated a self-organization process that included cellular differentiation. Interestingly, CD31^+^CD45^−^ cells showed elongated nuclei even in the absence of fluid shear stress. Nuclear elongation might have been the result of culturing cells in “mechanically stressed” fibrin gels that resemble granulation tissue. PBMC cultures without stromal cell support did not produce clusters with vasculogenic potential indicating that the presence and interactions of both MSC and PBMC were required for this response. Evidence for vascular development through mesenchymal cell-hematopoietic cell co-operation was provided by us in a previous study where we have shown that bone marrow-derived mononuclear cells embedded in fibrin matrices can form vascular structures through a dynamic self-organization process originating from cell clusters expressing CD34 and c-kit (Rüger et al., 2008). In the current study, a similar progenitor marker profile was found in intra-synovial cell clusters as well as in synovial tissue-derived mononuclear cell clusters external from the cultured tissue fragments, where CD34^+^ vascular sprouts emanated in close association with c-kit^+^ cells. C-kit is expressed by a subpopulation of MSC in the highly vascularized adipose tissue showing increased proliferative activity and self-renewal capacity (Blazquez-Martinez et al., 2014), and *in vivo* vascular niches of synovial tissues from patients with RA and OA contain STRO-1 positive MSC found in clusters together with EPC as demonstrated by our group previously (Rüger et al., 2004). It is possible that EPC located in areas of new vessel formation within the synovial tissue migrated into the fibrin matrix or alternatively were generated *in situ* and either assisted in new capillary sprouting by angiogenesis, or in co-operation with MSC initiated *de novo* formation of vessel structures by vasculogenesis. Supporting this possibility are recent studies showing that EPC can form vessel-like structures in fibrin matrices when co-cultured with adipose-derived MSC (Holnthoner et al., 2015), and that fibrin is a suitable matrix for the growth, differentiation and angiogenesis capability of EPC enhancing cell retention and paracrine cytokine release relevant for neovascularization (Barsotti et al., 2011).

A fundamental step in vascular morphogenesis is the stabilization of newly formed vascular structures through pericyte recruitment and basement membrane matrix deposition including collagen IV production (Stratman et al., 2009). In our study, neo-vessels formed during explant culture produced a Col-IV^+^ basement membrane and where surrounded by cells expressing Col-IV. Similarly, in co-cultures of MSC with PBMC, CD31^+^ endothelial cells originating from cell clusters were closely associated with Col-IV^+^ stromal cells and a Col-IV^+^ extracellular matrix scaffold. Our finding that MSC produce large amounts of Col-IV was particularly interesting as vascular basement membrane assembly (including the deposition of Col-IV) coincides with pericyte recruitment (Stratman et al., 2009), and confirms the association of MSC with a subset of pericytes (Crisan et al., 2008). The development of a vascular lumen is essential for the formation of a functional vasculature. In the presented model of inflammatory neovascularization, caspase-3^+^ apoptotic cells were exclusively found in the core of neo-vessels that developed within and outside of the embedded synovial explant tissues suggesting that in this static setting, lacking blood circulation, lumen formation occurred through cavitation. At present it is generally thought that cord- or cell-hollowing—the formation of a lumen by creation of fluid-filled spaces between cells or within single cells (Sigurbjörnsdóttir et al., 2014)—are the major mechanisms of vascular lumen formation. However, it has also been shown that during rat glomerular development capillary lumen formation relies on apoptosis (Fierlbeck et al., 2003) and recent data indicate that apoptosis is also involved in lumen formation during placental vasculogenesis (Tertemiz et al., 2005). It is possible that both mechanisms contribute to lumen formation in a context-dependent fashion.

MSC actively interact and co-operate with immune cells and have the ability to adopt different phenotypes in response to sensing an inflammatory environment thereby regulating tissue homeostasis (Bernardo and Fibbe, 2013). Here we show that MSC in a 3D fibrin environment respond to paracrine inflammatory signals such as TNFα and IFNγ with increased development of STRO-1^+^ filopodial protrusions, structures reported to act as antennae for cells to probe their environment (Galbraith et al., 2007), and involved in numerous cellular processes, including wound healing, adhesion to the extracellular matrix and guidance toward chemoattractants (Mattila and Lappalainen, 2008). MSC filopodia may have promoted physical contact and direct cell-cell communication between stromal cells and immune cells in MSC-PBMC co-cultures thereby also influencing the cell fate of emerging cellular entities. STRO-1 has been classically defined as mesenchymal stem cells marker, but more recently has also been found in endothelial cells (Ning et al., 2011). In our co-culture model STRO-1 positivity was associated with both stromal- and endothelial cells. While MSC-PBMC co-cultures generated cells of endothelial phenotype co-expressing CD31 and STRO-1, MSC under paracrine inflammatory stimulation but without immune cell contact exclusively expressed the mesenchymal marker STRO-1 and were CD31 negative. These results suggest that MSC-PBMC contact was necessary for the generation of cells of this mixed phenotype indicating that STRO-1 expression might be associated with vascular development in the presence of immune cells, e.g., during inflammatory processes.

The paracrine signature of the inflammatory niche environment is dependent on and influenced by the interaction and co-operation of niche cells and in turn affects cell fate decisions and structural remodeling. The results of the co-culture experiments in fibrin-based scaffolds indicate that the communication of inflammatory cells with MSC induce a dynamic increase of pro-angiogenic factors concurrent with the generation of cells participating in neovascularization. Cluster formation and cellular re-structuring toward neo-vessel growth was associated with increased release of VEGF, IL-6 and G-CSF when MSC were co-cultured with PBMC in the 3D environment. Direct and indirect MSC-PBMC interactions seemed to be essential for their secretion, as none of the cytokines were produced at detectable levels by PBMC cultured without stromal support. MSC secreted considerable (but lower) levels of VEGF in the absence of PBMC, and VEGF production in fibrin gels was augmented by pro-inflammatory cytokines supporting previous reports (Kagiwada et al., 2008; Yang et al., 2017). When MSC were co-cultured with PBMC, VEGF secretion increased 11-fold compared to control MSC mono-cultures, and this increase was cell-contact dependent, as VEGF release was significantly decreased in transwell co-cultures dropping to levels comparable to those produced by MSC stimulated by TNFα and IFNγ. The dramatic increase of VEGF coincided with the formation of vasculogenic cell clusters with emerging EC-like cells in the fibrin matrices suggesting that both paracrine and physical stromal cell-immune cell co-operation mechanisms were responsible for cells of endothelial phenotype to arise. The cell-tracking experiments of this study provide evidence for such close physical contact. Paracrine signals from MSC, e.g., VEGF, might have promoted the *in situ* growth of EPC in MSC-PBMC co-cultures leading to further increase of VEGF levels and subsequent development of endothelial cells. Interestingly, *early EPC*—a hematopoietic subset of EPC secreting angiogenic cytokines including VEGF and showing *in vivo* vasculogenic capacity—can be obtained by short-term culture of PBMC on fibronectin-coated plates in endothelial cell culture medium containing VEGF (Hur et al., 2004). These findings corroborate our line of thought that paracrine signals from MSC (i.e., VEGF) may have promoted *in situ* EPC differentiation in the fibrin matrix, an environment that provides optimal support for the growth and differentiation of peripheral blood EPC (Barsotti et al., 2011). While VEGF and IL-6 were secreted by MSC even in the absence of PBMC, albeit at levels much lower than in co-culture, G-CSF was detected only in the presence of both MSC and PBMC, and the G-CSF release was largely dependent on direct cell-cell communication, as contact-prevention between MSC and PBMC led to greatly reduced secretion levels. Growth factor secretion by explant cultures demonstrated striking similarities to the cytokine pattern released by MSC-PBMC co-cultures (i.e., increased secretion of G-CSF, VEGF, and IL-6) suggesting related cellular interactions in both culture settings. Our data on the cytokine profile of cultured synovial explant tissues are in line with published clinical data showing that IL-6 is elevated in the synovial fluid and serum of patients with RA and OA (Houssiau et al., 1988; Pearson et al., 2017), and are consistent with previous reports demonstrating that both IL-6 and G-CSF can stimulate new vessel growth by promoting the production of VEGF (Cohen et al., 1996; Ohki et al., 2005). Furthermore, the paracrine signals produced by cultured synovial tissues correspond to prior work where high G-CSF concentrations were found in the serum and synovial fluid of patients with RA and OA (Nakamura et al., 2000; Mabey et al., 2014), and where raised VEGF levels in RA and OA patients correlated with disease severity (Ballara et al., 2001; Mabey et al., 2014), demonstrating that the synovial tissue fragments can long-term retain their *in vivo* paracrine signature in the 3D culture setting. Recent data suggest that the cross-talk between MSC and immune cells could be the basis of a vicious circle driving RA chronicity and progression (De Bari, 2015). High G-CSF levels in RA could be a result from such a cross-talk, and this possibility is supported by our co-culture data showing that MSC interaction with PBMC in fibrin matrices leads to enhanced G-CSF secretion with concomitant generation cells participating in neo-vessel formation. We therefore believe that the co-culture model approximates important aspects of the *in vivo* co-operative mechanisms between stromal cells and immune cells in the context of a pro-inflammatory environment. However, due to ethical reasons we were not able to perform control experiments using healthy synovial tissue posing an obvious limitation in this study. Even so, vascular outgrowth was dependent on the presence of inflammatory cells. Tissue pieces lacking inflammatory cells and thus leukocyte egress did not generate vascular outgrowth. Thus, such tissue pieces might be considered a surrogate control supporting our findings. Therefore, despite this limitation, we believe that the *in vitro* 3D culture system and the two presented models add important aspects to complex *in vivo* regenerative processes at the tissue level and may contribute to better understanding of complex cell-cell interactions underlying chronic inflammatory diseases.

## Concluding remarks

In conclusion, our study has shown that the 3D fibrin matrix system represents a highly suitable biologically relevant environment for the long-term culture of tissue fragments and provides a useful model to investigate context-dependent new vessel formation during pathological inflammatory processes. The *in vitro* explant tissue culture model offers a disease-related platform to integrate complex cell-cell and cell-matrix interactions with associated paracrine signaling patterns in a setting that mimics the *in vivo* situation. Therefore, it might provide a suitable tool to decode microenvironmental cues that promote chronic inflammation and disease in order to find new treatment options. The 3D co-culture model is equally useful for unveiling novel mechanisms of cellular co-operation during regenerative processes and chronic inflammatory diseases emphasizing the relevance of stromal cell-immune cell interactions thereby adding new aspects to the principles underlying self-organization. It can help to elucidate distinct cellular behavior patterns in a physiological 3D environment that—dependent on the specific combination of environmental cues—physically and chemically influences cell migration, differentiation and survival.

## Author contributions

BMR designed the work, generated, analyzed and interpreted data and wrote the paper. TB generated, analyzed and interpreted data and revised the work for intellectual content. AG and BK supported the study by providing synovial tissue; MBF and JMB revised the work for intellectual content.

### Conflict of interest statement

The authors declare that the research was conducted in the absence of any commercial or financial relationships that could be construed as a potential conflict of interest. The reviewer SC and handling Editor declared their shared affiliation.
