# Supplementary material for: Vascular Morphogenesis in the Context of Inflammation: Self-Organization in a Fibrin-Based 3D Culture System
Source: Front Physiol. 2018 Jun 5;9:679. doi: 10.3389/fphys.2018.00679 (PMC5996074; doi:10.3389/fphys.2018.00679)
Supplement: Supplementary file 1 [file Image_1.PDF]

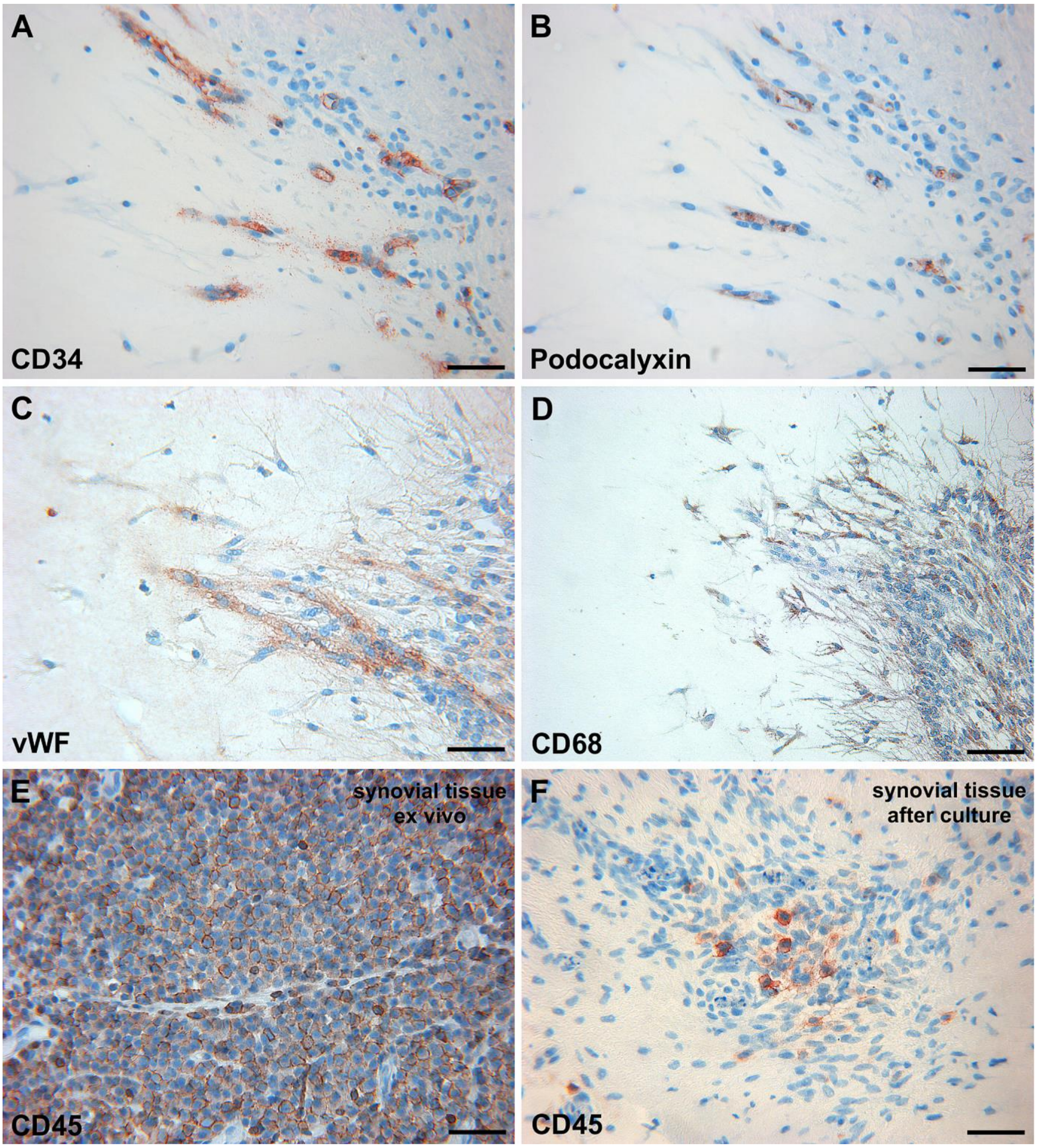

Supplemental Figure 1: <u>Characterization of synovial tissue outgrowth.</u> Neo-vessels formed during 3D fibrin matrix culture expressing the endothelial markers (A) CD34, (B) podocalyxin and (C) vWF. (D) Numerous $CD68^+$ cells are present around the neo-vessels. (A-D) Immunohistochemistry on consecutive paraffin sections of a representative day 20 RA explant culture sample. Scale bar 50 µm. (E) Paraffin section of freshly excised RA synovial tissue showing heavy infiltration with $CD45^+$ inflammatory cells. Scale bar 50 µm. (F) The corresponding adjacent tissue fragment after three weeks of culture in 3D fibrin matrix contains greatly reduced $CD45^+$ cells. Scale bar 50 µm.
