# Supplementary material for: Vascular Morphogenesis in the Context of Inflammation: Self-Organization in a Fibrin-Based 3D Culture System
Source: Front Physiol. 2018 Jun 5;9:679. doi: 10.3389/fphys.2018.00679 (PMC5996074; doi:10.3389/fphys.2018.00679)
Supplement: Supplementary file 2 [file Image_2.PDF]

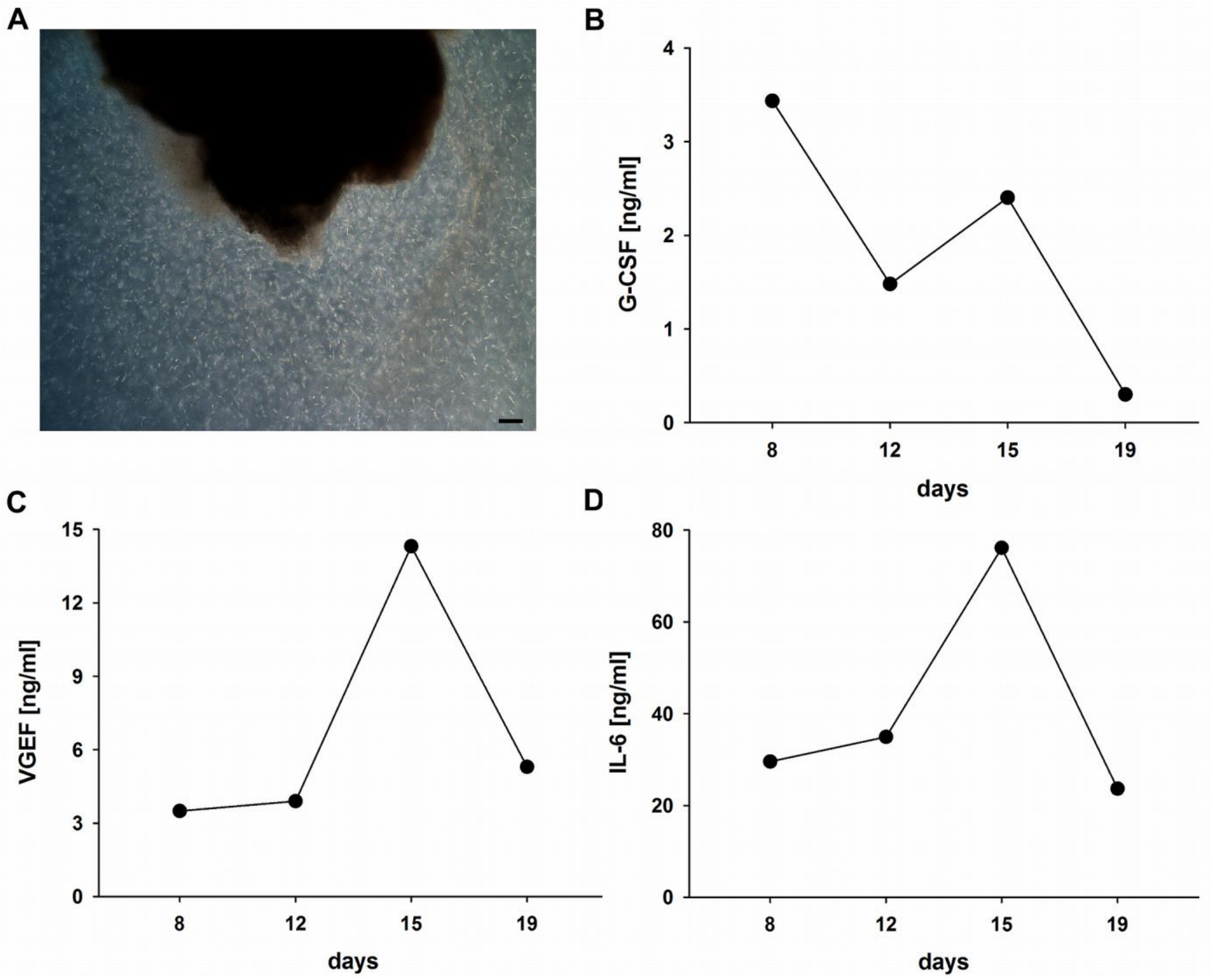

Supplemental Figure 2: <u>Synovial tissue without substantial inflammation does not show vascular outgrowth.</u> (A) Phase contrast microscopy image of a representative synovial tissue explant lacking mononuclear cell egress showing fibroblastic cell outgrowth with no signs of a vascular network on day 13. Scale bar, 200 µm. **<u>Kinetics of cytokine secretion.</u>** Determination of (B) G-CSF, (C) VEGF and (D) IL-6 by Bio-Plex cytokine assay in cell-free supernatants of the cultured explant sample shown in (A).
