# Supplementary material for: Vascular Morphogenesis in the Context of Inflammation: Self-Organization in a Fibrin-Based 3D Culture System
Source: Front Physiol. 2018 Jun 5;9:679. doi: 10.3389/fphys.2018.00679 (PMC5996074; doi:10.3389/fphys.2018.00679)
Supplement: Supplementary file 3 [file Image_3.pdf]

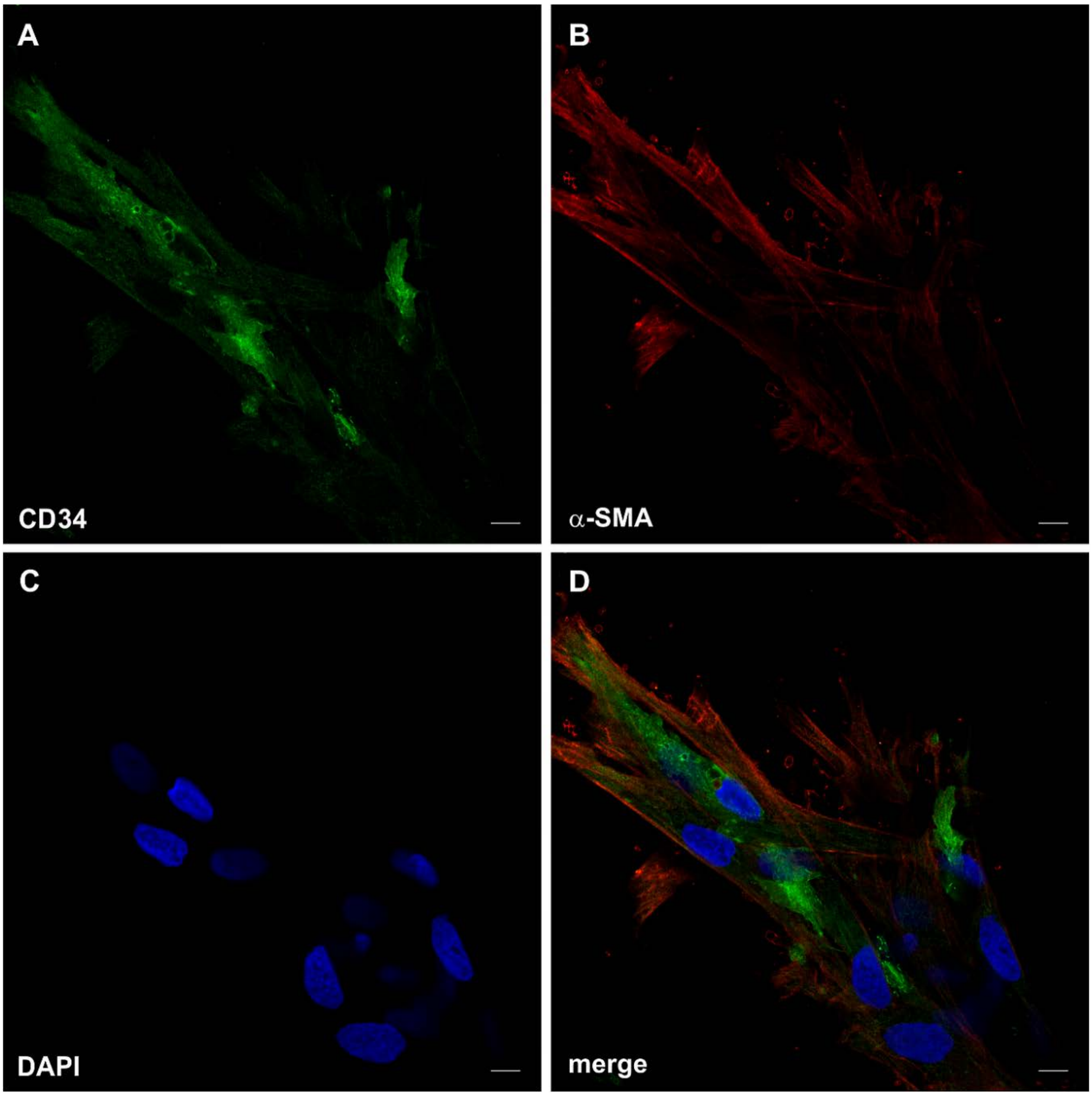

Supplemental Figure 3: **Neo-vessels are surrounded by mural cells.** A developing vascular sprout in the 3D fibrin matrix expressing (A) the endothelial marker CD34 and surrounded by (B) α-SMA$^+$ mural cells. (C) DAPI stain. (D) merge. CLSM images of intact 3D fibrin gel explant culture of OA synovial tissue on day 20. Scale bars 10 µm.
