# Supplementary material for: Vascular Morphogenesis in the Context of Inflammation: Self-Organization in a Fibrin-Based 3D Culture System
Source: Front Physiol. 2018 Jun 5;9:679. doi: 10.3389/fphys.2018.00679 (PMC5996074; doi:10.3389/fphys.2018.00679)
Supplement: Supplementary file 4 [file Image_4.pdf]

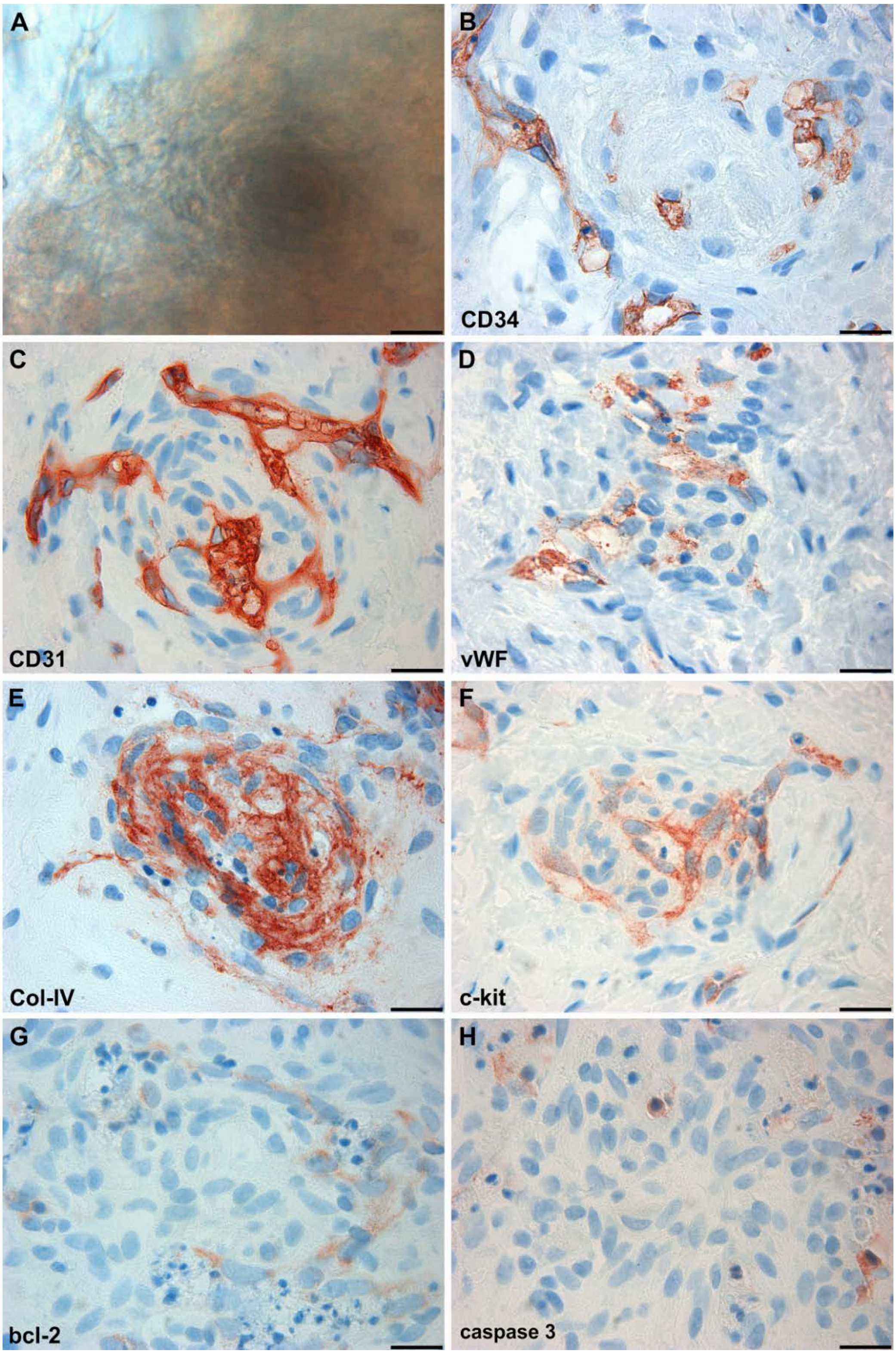


**Supplemental Figure 4:** **<u>Characterization of intra-synovial cell clusters</u>.** (A) Phase contrast microscopy image of a cell cluster located within a RA synovial tissue sample after 9 days of culture. Immature vascular sprouts emanating from the intra-synovial cell cluster express (B) CD34, (C) CD31, D) vWF and (F) c-kit. Cells expressing (G) bcl-2 are found in close vicinity to (H) active caspase 3 positive apoptotic cells. (E) Col-IV forms the structural scaffold of the cluster. (B-H) Immunohistochemistry on consecutive paraffin sections of RA synovial tissue cultured in 3D fibrin matrix for 9 days. Scale bars 20 µm.
