# Supplementary material for: Vascular Morphogenesis in the Context of Inflammation: Self-Organization in a Fibrin-Based 3D Culture System
Source: Front Physiol. 2018 Jun 5;9:679. doi: 10.3389/fphys.2018.00679 (PMC5996074; doi:10.3389/fphys.2018.00679)
Supplement: Supplementary file 5 [file Image_5.pdf]

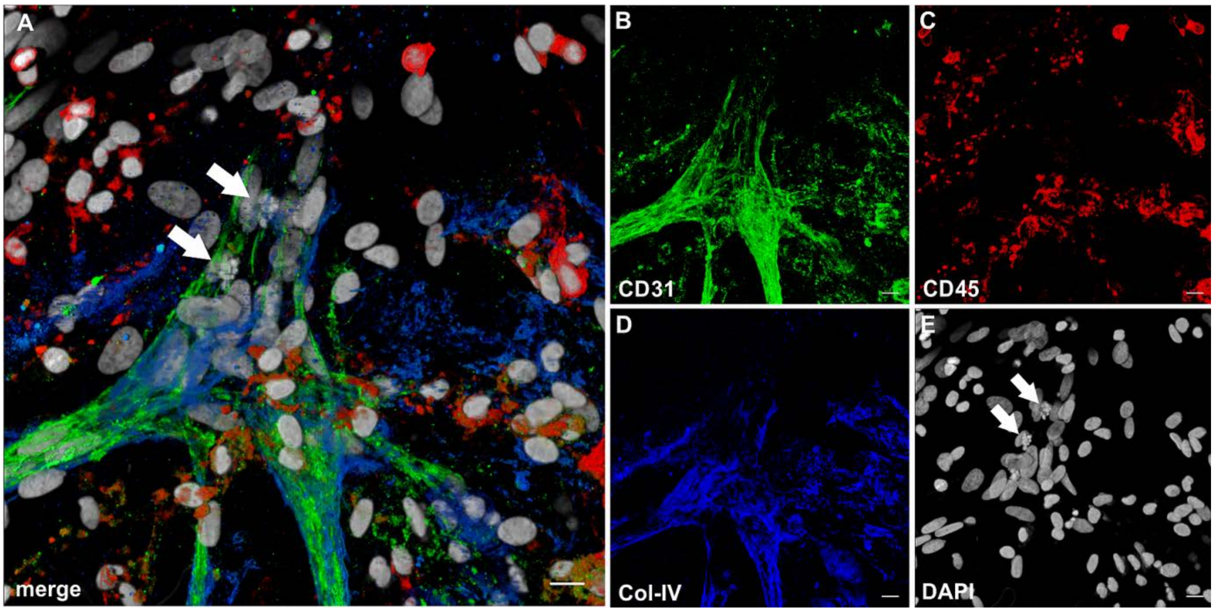

Supplemental Figure 5: **The core of vascular sprouts contains apoptotic cells.** Developing neo-vessel expressing (B) CD31, surrounded by (C) $CD45^{+}$ inflammatory cells and (D) $Col\text{-}IV^{+}$ cells. Apoptotic cells with condensed nuclei in the core of the vascular sprout (arrows) express CD31 and are $CD45^{-}$. (E) DAPI stain. (A) Merge. CLSM images of intact 3D fibrin gel explant culture of OA synovial tissue on day 21, collapsed z-stack. Scale bars 10 µm. (See also Supplemental Video 1, showing animated z-stack).
