# Supplementary material for: Vascular Morphogenesis in the Context of Inflammation: Self-Organization in a Fibrin-Based 3D Culture System
Source: Front Physiol. 2018 Jun 5;9:679. doi: 10.3389/fphys.2018.00679 (PMC5996074; doi:10.3389/fphys.2018.00679)
Supplement: Supplementary file 6 [file Image_6.pdf]

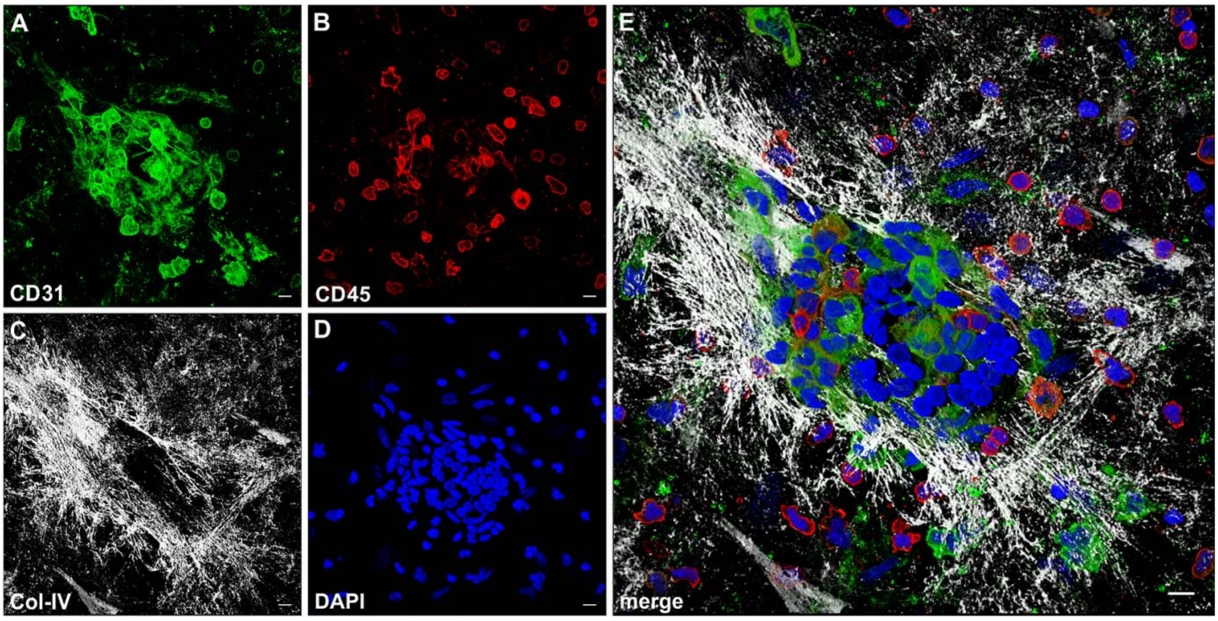

Supplemental Figure 6: <u>Cluster in 3D MSC-PBMC co-culture.</u> CLSM images of MSC-PBMC co-culture in 3D fibrin matrix on day 9. Cell cluster consisting of (A) CD31$^+$ cells (B) partially co-expressing CD45. The cluster is enwrapped in a scaffold of (C) Col-IV$^+$ stromal cells and matrix, and surrounded by CD45$^+$ leukocytes. (D) DAPI stain. (E) Merge. Collapsed z-stack. Scale bars, 10 µm. (See also corresponding animated z-stack, Supplemental Video 3)
