# Supplementary material for: Vascular Morphogenesis in the Context of Inflammation: Self-Organization in a Fibrin-Based 3D Culture System
Source: Front Physiol. 2018 Jun 5;9:679. doi: 10.3389/fphys.2018.00679 (PMC5996074; doi:10.3389/fphys.2018.00679)
Supplement: Supplementary file 7 [file Image_7.pdf]

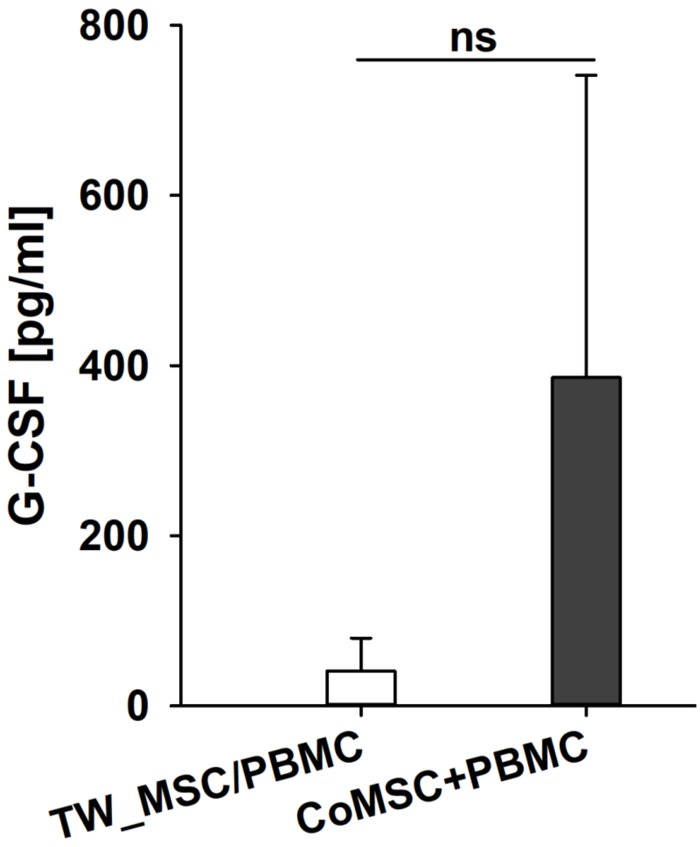

Supplemental Figure 7: **<u>G-CSF secretion in transwell experiments.</u>** Determination of G-CSF levels in cell-free supernatants of 3D co-cultures where MSC were physically separated from PBMC by a transwell insert with 0.4 µm pore size, and of corresponding 3D MSC-PBMC co-cultures on day six by ELISA (n=6).
