# Supplementary material for: Vascular Morphogenesis in the Context of Inflammation: Self-Organization in a Fibrin-Based 3D Culture System
Source: Front Physiol. 2018 Jun 5;9:679. doi: 10.3389/fphys.2018.00679 (PMC5996074; doi:10.3389/fphys.2018.00679)
Supplement: Supplementary file 8 [file Image_8.pdf]

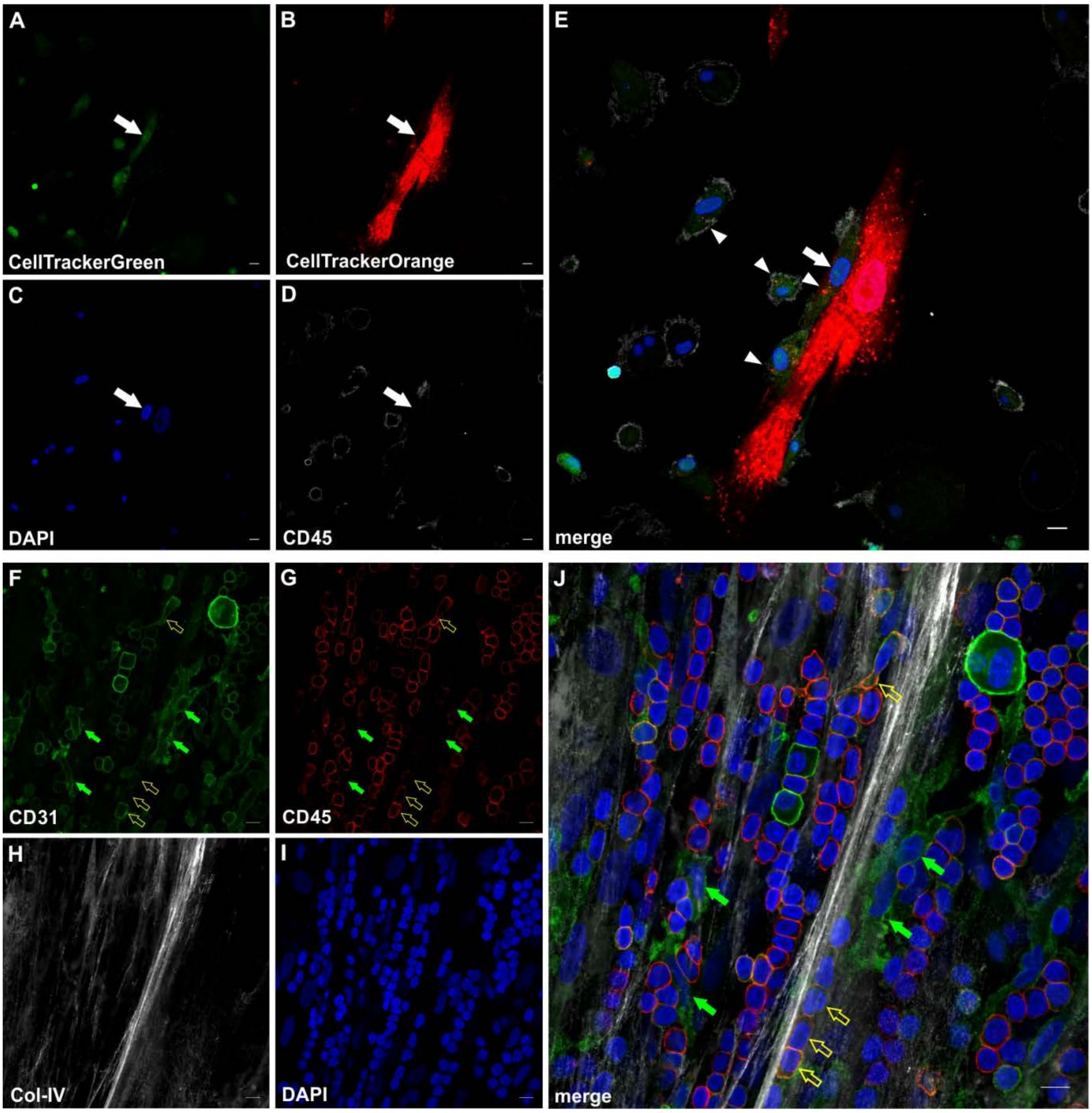

Supplemental Figure 8: **<u>Close contact of PBMC with MSC in 3D fibrin-based co-cultures.</u>** (A) PBMC labeled with Cell Tracker Green fluorescent probe and (B) MSC labeled with Cell Tracker Orange fluorescent probe showing (E) close physical contact of both cell types with each other. After fixation with 4% paraformaldehyde, co-cultures were stained with AF647-mouse anti-CD45 (Biolegend). PBMC express (D) CD45. (E) Note the very faint CD45 expression of the leukocyte closely attached to the stromal cell (arrow). Some CTGreen^{+}/CD45^{+} cells contain CTOrange fluorescent probe (arrow heads). (C) DAPI stain. (E) Merge. (A-E) Representative CLSM images of bone marrow MSC cultured with PBMC in 3D fibrin gels on day seven. Scale bars, 10 µm. In MSC-PBMC co-cultures (F) CD31^{+}/CD45^{-} cells (green arrows) together with (G) CD45^{+} leukocytes show ordered alignment to (H) Col-IV^{+} cells and matrix. (J) Merge. Chain-like arrangement of leukocytes with differential CD31/CD45 expression pattern alongside Col-IV^{+} cells and matrix (yellow open arrows). (I) DAPI stain. (F-J) CLSM images of MSC-PBMC co-culture in 3D fibrin matrix on day eight. Scale bars, 10 µm.
