# Supplementary material for: Vascular Morphogenesis in the Context of Inflammation: Self-Organization in a Fibrin-Based 3D Culture System
Source: Front Physiol. 2018 Jun 5;9:679. doi: 10.3389/fphys.2018.00679 (PMC5996074; doi:10.3389/fphys.2018.00679)
Supplement: Supplementary file 9 [file Image_9.PDF]

Supplemental Video 1: **Animated z-stack of Supplemental Figure 5:** The core of the developing neo-vascular sprout ($CD31^+$, green) contains apoptotic cells with condensed nuclei (pointed out by arrows in Supplemental Figure 5) expressing CD31. Apoptotic cells do not express CD45 (red). Collagen IV in blue. Nuclear DAPI stain in white. Scale bar 10 µm.
