# Supplementary material for: Vascular Morphogenesis in the Context of Inflammation: Self-Organization in a Fibrin-Based 3D Culture System
Source: Front Physiol. 2018 Jun 5;9:679. doi: 10.3389/fphys.2018.00679 (PMC5996074; doi:10.3389/fphys.2018.00679)
Supplement: Supplementary file 10 [file Image_10.PDF]

Supplemental Video 2: **Animated z-stack of the cluster shown in Figure 5: '**Cuddling' cluster cells in the lower plane of the gel show differential expression of CD45 (red) and CD31 (green). The cluster is enveloped by a Col-IV$^+$ meshwork (white) building a vault-like scaffold. Elongated CD31$^+$ cells partly expressing Col-IV emerge from the cluster. These endothelial-type cells appear to grow upwards towards the surface of the gel. Note the close physical contact of some CD45$^+$ leukocytes with Col-IV$^+$ stromal cells and the decrease/lack of CD45 in connection with nuclear elongation of CD31$^+$ cells. Nuclear stain DAPI (blue). Animated 16 µm-z-stack consisting of 33 consecutive images. Scale bar, 10 µm.
