# Supplementary material for: Vascular Morphogenesis in the Context of Inflammation: Self-Organization in a Fibrin-Based 3D Culture System
Source: Front Physiol. 2018 Jun 5;9:679. doi: 10.3389/fphys.2018.00679 (PMC5996074; doi:10.3389/fphys.2018.00679)
Supplement: Supplementary file 11 [file Image_11.PDF]

Supplemental Video 3: **Animated z-stack of cluster shown in Supplemental Figure 6E:** Col-IV$^+$ stromal cells (white) are building a vault-like scaffold under which cells with differential expression of CD31 (green) and CD45 (red) are present. Under this dome-like collagen shell, peripheral cluster cells close to the matrix vault show elongated nuclei. These cells are CD31$^+$, express CD45 very faintly or not at all and appear to build a sprout that leaves the collagen vault growing upwards into the gel. Nuclear stain DAPI (blue). Animated 10.5 µm-z-stack consisting of 16 consecutive images. Scale bar, 10 µm.
