# Supplementary material for: Vascular Morphogenesis in the Context of Inflammation: Self-Organization in a Fibrin-Based 3D Culture System
Source: Front Physiol. 2018 Jun 5;9:679. doi: 10.3389/fphys.2018.00679 (PMC5996074; doi:10.3389/fphys.2018.00679)
Supplement: Supplementary file 12 [file Image_12.PDF]

Supplemental Video 4: **Animated z-stack of Figure 7F:** MSC-PBMC co-culture on day 13 demonstrating elongated cells that co-express CD31 (green) and STRO-1 (red) emerging from Col-IV^{+} clusters (white). Some of these hybrid-type cells also show Col-IV expression. Round CD31^{+} cells are STRO-1^{-}. STRO-1^{+}CD31^{-} MSC show long cell protrusions. Nuclear stain DAPI (blue). Animated 30 µm-z-stack consisting of 21 consecutive images Scale bar, 20 µm.
